# Widespread Distribution of Highly Adapted *Bradyrhizobium* Species Nodulating Diverse Legumes in Africa

**DOI:** 10.3389/fmicb.2019.00310

**Published:** 2019-02-22

**Authors:** Sanjay K. Jaiswal, Felix D. Dakora

**Affiliations:** ^1^Department of Chemistry, Faculty of Science, Tshwane University of Technology, Pretoria, South Africa

**Keywords:** biogeography, cowpea, groundnut, Bambara groundnut, wild legumes, soil factors, novel species

## Abstract

*Bradyrhizobium* is one of the most cosmopolitan and diverse bacterial group nodulating a variety of host legumes in Africa, however, the diversity and distribution of bradyrhizobial symbionts nodulating indigenous African legumes are not well understood, though needed for increased food legume production. In this review, we have shown that many African food legumes are nodulated by bradyrhizobia, with greater diversity in Southern Africa compared to other parts of Africa. From a few studies done in Africa, the known bradyrhizobia (i.e., *Bradyrhizobium elkanii, B. yuanmingense*) along with many novel *Bradyrhizobium* species are the most dominant in African soils. This could be attributed to the unique edapho-climatic conditions of the contrasting environments in the continent. More studies are needed to identify the many novel bradyrhizobia resident in African soils in order to better understand the biogeography of bradyrhizobia and their potential for inoculant production.

## Introduction

Globally, farmers depend on N fertilizers for increased crop yields (Crews and Peoples, 2004). Even then, sub-Saharan Africa currently uses the least chemical fertilizers compared to other regions (Ruben et al., 2007), due largely to high cost of these inputs for resource-poor farmers, their inaccessibility and potential to pollute the environment, as well as poor infrastructure (Dakora and Keya, 1997; Chianu et al., 2011). These factors together have limited the use of N fertilizers in African agriculture, even though there is a growing demand for increased crop production to feed the growing population (Dakora and Keya, 1997; Aticho et al., 2011; Chianu et al., 2011; Lal and Stewart, 2013). To feed the expected 2.5 billion people by 2050, Africa will need to roughly double its agricultural production. Food production is, however, associated with environmental pollution from anthropogenic activity. For example, the current level of industrialization has been achieved with 70% of the water used by people, 80% of deforestation worldwide, 70% loss of biodiversity and nearly one-quarter of the total greenhouse gas emissions (Tilman et al., 2011; Kessy et al., 2016). The world therefore urgently needs to increase agricultural productivity in a sustainable and environmentally-friendly manner. The large increase in population observed today would require at least 20% increase in crop yields in order to meet global food/nutritional security (Evans and von Caemmerer, 2011). This in turn may require the expansion of agricultural activity into marginal and/or non-arable land in order to feed the growing human population (Hungria and Vargas, 2000).

Africa is one of the major centers of legume diversity in the world, and is therefore home to a large number of indigenous legume species (Sprent, 2009; Beukes et al., 2013). In Africa, grain legumes remain the key source of dietary protein and starch (Bezner Kerr et al., 2007). Legumes also serve as high protein feed for livestock production, and biofertilizer for maintaining soil productivity. Therefore, legume inclusion in cropping systems has the potential to increase crop yields, which are currently very low at farm level in Africa when compared to the rest of the world (Akibode, 2011). Furthermore, N_2_ fixation in legumes can provide economic, environmental and agronomic benefits to farmers in Africa where socio-economic conditions are poor (Chianu et al., 2011; Siddique et al., 2011). It is thus not surprising that BNF is the most important biological process on earth after photosynthesis (Unkovich et al., 2008) as it not only reduces fossil energy use globally, but also sustainably promotes increased agricultural yields without environmental damage. BNF is, however, influenced by many factors, which include geographic location, soil type, host–plant genotypes, and the rhizobial symbionts (Jaiswal et al., 2017).

Of the proteobacteria, *Bradyrhizobium* is an ancestral symbionts and highly cosmopolitan in terms of its distribution as a free-living bacterium in different habitats and in symbiosis with diverse leguminous hosts (Sprent et al., 2017). Like other N_2_-fixing microsymbionts, *Bradyrhizobium* exhibits a bipartite life cycle which alternates between the free-living state in soils and as a symbiotic partner inside root nodules of legumes. Its chromosome is also bipartite in nature, with the main chromosomal genes being largely expressed under free-living conditions, and symbiosis genes expressed *in planta*. The plasmids or genomic island of root-nodule bacteria harbor all the symbiotic genes, which are usually transmitted vertically, or horizontally between different chromosomal backgrounds.

The interaction of legumes with root-nodule bacteria has become a model for dissecting the molecular conversation between rhizobial strains and their host plants, and is the best understood mutualistic relationship, especially when compared to other systems such as the mycorrhizal symbiosis with land plants and the Frankia symbiosis with actinorhizal plants.

While we currently have a clearer understanding of the molecular signals involved in the early stages of nodule formation, these insights get masked by the diversity of legumes and their rhizobia, as well as the ecological niches that interact to produce root nodules. As a result, we do not know how natural selection shapes each partner, as well as the extent to which the interaction can vary depending on intrinsic and extrinsic factors.

**FIGURE 1:**
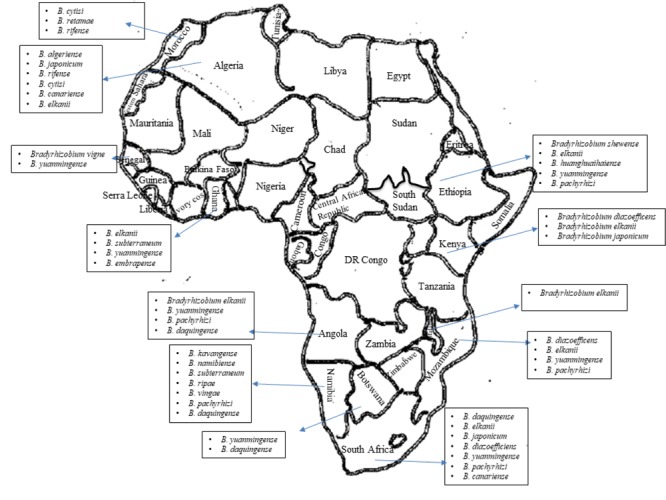
Presence of diverse bradyrhizobial population in African soils.

Also, we still do not understand the community structure and composition of bradyrhizobia found in African soils, even though we know that only certain types of bradyrhizobia are known to nodulate the diverse range of legumes grown in Africa (see Figure 1). Studies based on a few African countries seem to suggest that the number, species and strains of bradyrhizobial populations found in African soils are individually and collectively much greater than that shown in Figure 1. Only future studies will unravel the scale of novel bradyrhizobia resident in African soils that are waiting to be discovered and identified using modern molecular technologies. This review summarizes the status of legume root nodulation by bradyrhizobia in Africa.

## Legumes Nodulated by *Bradyrhizobium* Species in African Soils

Reports on legume nodulation by *Bradyrhizobium* in Africa are rather scanty in terms of (i) the diversity of legumes studied (ii) the types and numbers of bradyrhizobial microsymbionts found to nodulate legumes in Africa, and (iii) the huge number of unknown bradyrhizobial isolates waiting to be delineated and identified. In our laboratory alone, we have studied and reported on the nodulation of cowpea (*Vigna unguiculata* L. Walp), Bambara groundnut (*Vigna subterranean* L. Verdc), Kersting’s bean (*Macrotyloma geocarpum* Harns), groundnut (*Arachis hypogaea* L.), common bean (*Phaseolus vulgaris* L.) and soybean (*Glycine max* L. Merr) in different agro-ecologies of selected African countries, which include Ghana, South Africa, Ethiopia, Mozambique, Zambia, Swaziland, and Mali. This review is a summary of our work together with those of others reported from across Africa. In this study, we intend to summarize data on *Bradyrhizobium* nodulation of cowpea, groundnut, soybean and Bambara groundnut together with data on nodulation of other wild African legumes by bradyrhizobia.

## Diversity of Microsymbionts Nodulating Indigenous *Vigna* Species in Africa

Cowpea (*Vigna unguiculata* L. Walp.) is a major food crop in Africa, its organs such as leaves, green pods and grain are eaten as a source of protein (Pule-Meulenberg et al., 2010). It is a promiscuous legume that is often used to trap microsymbionts in soil during diversity studies (Guimarães et al., 2012; Costa et al., 2013; Jaramillo et al., 2013; Grönemeyer et al., 2015a, 2016; Chidebe et al., 2018). This legume can establish effective symbioses with diverse bacterial species belonging to the genera *Rhizobium* and *Bradyrhizobium* (Wade et al., 2014; Chidebe et al., 2018). As a result, cowpea has the ability to grow in diverse environments where other legumes may fail to survive. The cowpea symbiosis can meet up to 96% of the plant’s N requirement, and contribute substantially to the N needs of subsequent cereal crops, especially where the soils are nutrient-poor (Peoples et al., 2009; Belane and Dakora, 2010). Some studies have shown that cowpea can derive as much as 66% or more of its N nutrition from symbiotic fixation in Botswana (Pule-Meulenberg and Dakora, 2009), up to 99% in Ghana (Naab et al., 2009), and 15–56% in Zimbabwe (Ncube et al., 2007). These differences in N obtained from symbiosis can be attributed to the functional diversity among the *Bradyrhizobium* strains nodulating cowpea in Africa (Pule-Meulenberg et al., 2010; Grönemeyer et al., 2014; Chidebe et al., 2018).

Earlier studies have identified *Bradyrhizobium* as the dominant microsymbiont nodulating cowpea in Botswana, Ghana, South Africa (Steenkamp et al., 2008; Pule-Meulenberg et al., 2010); Senegal (Wade et al., 2014), Mozambique (Chidebe et al., 2018), Ethiopia (Degefu et al., 2017), and Angola, as well as Namibia (Grönemeyer et al., 2014). Cowpea microsymbionts have a broad host nodulation range (Mpepereki et al., 1996). However, Africa is vast and agro-ecologically divergent, therefore many more studies are needed on the diversity and phylogeny of cowpea-nodulating microsymbionts in the continent, the center of its origin and diversification. The characterization of native rhizobia from the different geographic regions of Africa is likely to increase our understanding of their contribution to ecosystem functioning and thus help to unravel the factors shaping microsymbiont diversity and distribution in African soils.

Perhaps because Africa is the origin of cowpea, there is a vast diversity of cowpea rhizobia present in African soils (Steenkamp et al., 2008; Pule-Meulenberg et al., 2010; Wade et al., 2014; Degefu et al., 2017; Chidebe et al., 2018). This would be consistent with the view that the sites of origin of legumes tend to coincide with the centers of diversity of their associated microsymbionts needed for nodule formation and N_2_ fixation (Puozaa et al., 2017). Furthermore, it has been argued that because Brazil and the Southern African region share similar climatic conditions (Chibeba et al., 2017) is probably the reason why only Southern Africa and Brazil have added more novel *Bradyrhizobium* species to that genus nodulating cowpea. The first cowpea-nodulating *Bradyrhizobium* was identified in the Amazon soils of Brazil, and classified as *Bradyrhizobium manausense* (Silva et al., 2014). Later, two *Bradyrhizobium* species [i.e., *Bradyrhizobium kavangense* (Grönemeyer et al., 2015b) and *B. vignae* (Grönemeyer et al., 2016)] were isolated from cowpea root nodules in Southern Africa (Namibia and Angola, respectively). More recently, *B. brasilense* (da Costa et al., 2017) was also identified in Brazilian soils (Table 1).

**Table 1:** Morph-physiological and general characteristics of *Bradyrhizobium* type strains.

Species name	Legume host	Origin	Tolerance to NaCl	Temperature range for growth (°C)	pH	Growth rate (h) and colony size (mm)	Reference
*B. americanum*	*Centrosema macrocarpum*	Venezuela	<1%	15–35	6–8	–(<1)	Ramírez-Bahena et al., 2016
*B. arachidis*	*Arachis hypogaea*	China	< 1%	20–30	6–8	8.8(-)	Wang R. et al., 2013
*B. algerians*	*Retama sphaerocarpa*	Algeria	1%	28–30	4–8	14–23(-)	Ahnia et al., 2018
*B. betae*	*Beta vulgaris*	Spain		28–30	7.5		Rivas et al., 2004
*B. brasilense*	*Vigna unguiculata*	Brazil			4–10		da Costa et al., 2017
*B. cajani*	*Cajanus cajan*	Dominican Republic		18–37	5–9		Araújo et al., 2017
*B. canariense*	*Chamaecytisus proliferus*	Spain		28–30	4–7		Vinuesa et al., 2005
*B. centrolobii*	*Centrolobium paraense*	Brazil	<1%	20–36	5–11	–(3–4)	Michel et al., 2017
*B. centrosemae*	*Centrosema molle*	Venezuela	1%	10–37	4.5–7.5	–(<1)	Ramírez-Bahena et al., 2016
*B. cytisi*	*Cytisus villosus*	Morocco	<0.25	14–30	6–8		Chahboune et al., 2011
*B. daqingense*	*Glycine max*	China	1%	28–37	6–9	10 (1)	Wang J.Y. et al., 2013
*B. denitrificans*	*-*	Germany		28–41			Van Berkum et al., 2006
*B. diazoefficiens*	*Glycine max*	Japan	<1%	28	4.8–6.8	–(1.2–1.5)	Delamuta et al., 2013
*B. elkanii*	*Glycine max*	United States					Kuykendall et al., 1992
*B. embrapense*	*Desmodium heterocarpon*	Brazil	<1%	28–37	4.5–8.0	7.49(-)	Delamuta et al., 2015
*B. erythrophlei*	*Erythrophleum fordii*	China	1%	4–60	5–8	14.6 (<1)	Yao et al., 2015
*B. ferriligni*	*Erythrophleum fordii*	China	1%	4–45	5-8	13.1 (1)	Yao et al., 2015
*B. forestalis*	Amazon legume tree	Brazil	<1%	15–37	4–10	–(>1)	da Costa et al., 2018
*B. ganzhouense*	*Acacia melanoxylon*	China	3%	4–37	5–12	–(1–2)	Lu et al., 2014
*B. guangdongense*	*Arachis hypogaea*	China	1%	15–28	5–7	16.94 (1–2-)	Li et al., 2015
*B. guangxiense*	*Arachis hypogaea*	China	1%	15–28	5–7	13.49(1–2)	Li et al., 2015
*B. huanghuaihaiense*	*Glycine max*	China	<1%	10–37	6–9	7–9 (1)	Zhang et al., 2012
*B. icense*	*Phaseolus lunatus*	Peru	1%	28	5.5–10	11–12(1)	Durán et al., 2014a
*B. ingae*	*Inga laurina*	Brazil, Roraima	<0.5%	15–32	4–8	9.5(1)	da Silva et al., 2014
*B. iriomotense*	*Entada koshunensis*	Japan, Okinawa	<1%	15–32	4.5–9.0		Islam et al., 2008
*B. japonicum*	*Glycine max*	Japan	<2%	25–30	3.5–9.0	–(<1)	Jordan, 1982
*B. jicamae*	*Pachyrhizus erosus*	Honduras	1%	5–32	6–8	–	Ramírez-Bahena et al., 2009
*B. kavangense*	*Vigna unguiculata*	Namibia, Kavango	<1%	28–38	5–9	7.5 (0.2–0.8)	Grönemeyer et al., 2015b
*B. lablabi*	*Lablab purpureus*	China	<1%	10–37	5–10	10–12(<1)	Chang et al., 2011
*B. liaoningense*	*Glycine max*	China	<1%	25–30	7.45–8.08	–(0.2–1)	Xu et al., 1995
*B. lupini*	*Lupinus angustifolius*	United States	<1%	28	7		Peix et al., 2015
*B. manausense*	*Vigna unguiculata*	Brazil	<0.05	15–32	4–8	7.8 (1)	Silva et al., 2014
*B. macuxiense*	*Centrolobium paraense*	Brazil	<1%	20–36	5–11	–(3–4)	Michel et al., 2017
*B. mercantei*	*Deguelia costata*	Brazil	<1%	28–30	4.5–8	–(<1)	Helene et al., 2017
*B. namibiense*	*Lablab purpureus*	Namibia		–			Grönemeyer et al., 2017
*B. neotropicale*	*Centrolobium paraense*	Brazil, Roraima	<1.5%	15–37	4–10	10.8 (1)	Zilli et al., 2014
*B. oligotrophicum*	Rice	–	<0.5	28–37	–	–	Ramírez-Bahena et al., 2013
*B. ottawaense*	*Glycine max*	Canada	<1%	20	5–10	12–13(<1)	Yu et al., 2014
*B. pachyrhizi*	*Pachyrhizus erosus*	Honduras	<1%	5–37	4.5–8	–	Ramírez-Bahena et al., 2009
*B. paxllaeri*	*Phaseolus lunatus*	Peru	1%	28–37	5.5–10	11–12(1)	Durán et al., 2014a
*B. retamae*	*Retama sphaerocarpa*	Morocco	<1%	14–30	6–8	–(<1)	Guerrouj et al., 2013
*B. rifense*	*Cytisus villosus*	Morocco	<1%	14–30	4.5–8.0	(<1)	Chahboune et al., 2012
*B. ripae*	*Indigofera rautanenii*	Namibia					Bünger et al., 2018
*B. sacchari*	*Sugar cane*	Brazil	<0.5	20–37	5–12	–(1.5–2)	de Matos et al., 2017
*B. shewense*	*Erythrina brucei*	Ethiopia	0.5%	15–30	5–10	–(1–2)	Aserse et al., 2017
*B. stylosanthis*	*Stylosanthes guianensis*	Brazil	<1%	28	4.5–8.0	(1–1.34)	Delamuta et al., 2016
*B. subterraneum*	*Vigna subterranea*	Namibia, Kavango	<1%	28–37	5–9	8 (0.2-1)	Grönemeyer et al., 2015a
*B. tropiciagri*	*Neonotonia wightii*	Brazil	<1%	28	4.5–8.0	7.42(-)	Delamuta et al., 2015
*B. valentinum*	*Lupinus mariae-josephae*	Spain	<1%	14–30	4–10	–(<2)	Durán et al., 2014b
*B. vignae*	*Vigna unguiculata*	Namibia	<1%	28–40	5–9	8 (0.2–1)	Grönemeyer et al., 2016
*B. viridifuturi*	*Centrosema pubescens*	Brazil	<1%	28	4.5–7.0	(0.5–1.5)	Helene et al., 2015
*B. yuanmingene*	*Lespedeza cuneata*	China	<1%	25–30	6.5–7.5	9.5–16(<1)	Yao et al., 2002

**FIGURE 2:**
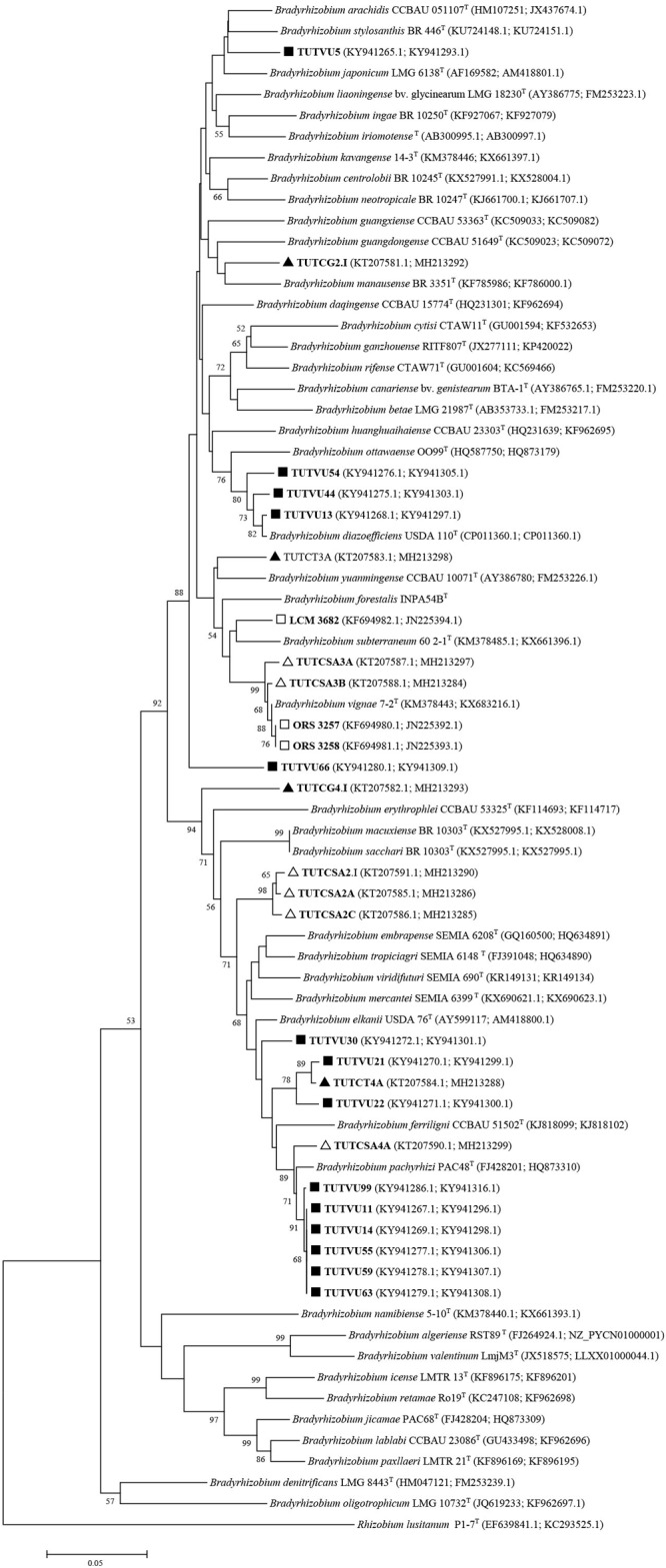
Neighbor-joining molecular phylogenetic analysis of cowpea nodulating rhizobia from ▲Ghana, □ Senegal, ■ Mozambique, and △ South Africa based on concatenated *glnII* + *gyrB* (495 bp) sequences with type strains of *Bradyrhizobium* species. The evolutionary history was inferred by using the Neighbor-joining method based on the Kimura 2-parameter model. The scale bar indicates the number of substitutions per site. The percentage of trees in which the associated taxa clustered together in the bootstrap test (1000 replicates) are shown next to the branches. All positions containing gaps and missing data were eliminated. Evolutionary analyses were conducted in MEGA6.

**FIGURE 3:**
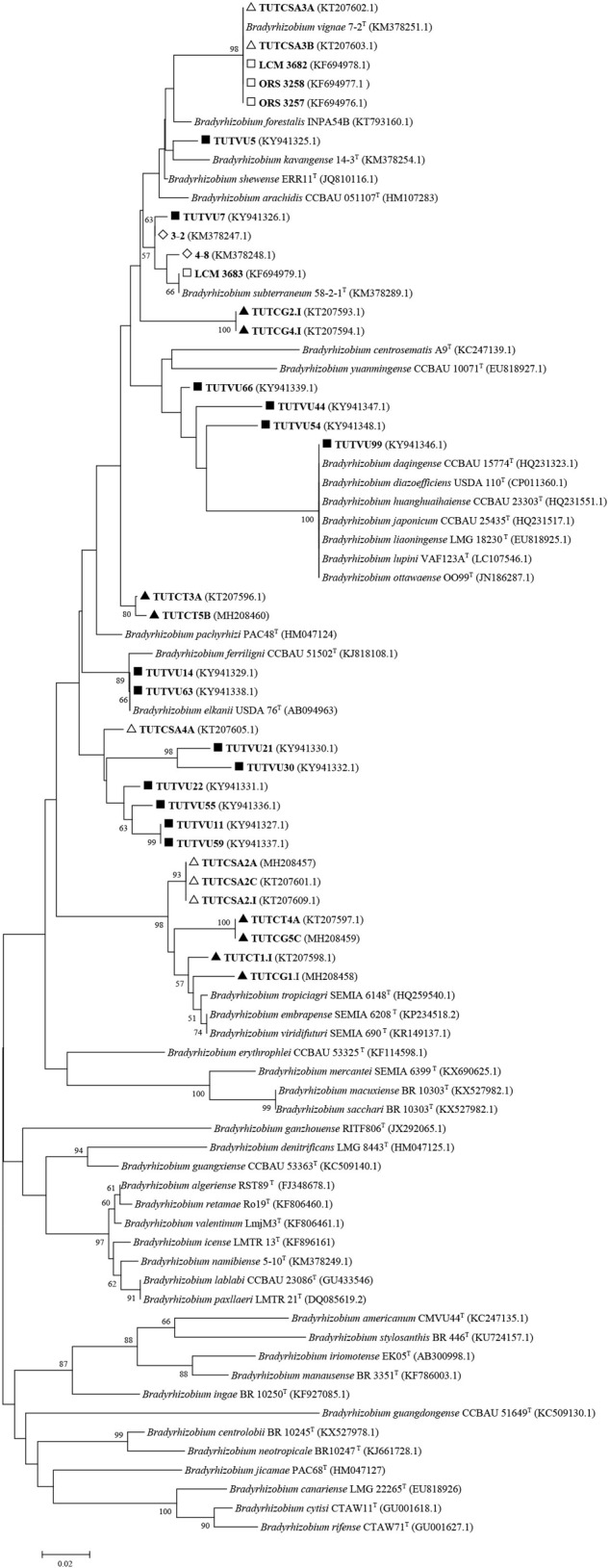
Neighbor-joining molecular phylogenetic analysis of cowpea nodulating rhizobia from ▲Ghana, □ Senegal, ■ Mozambique,◇ Namibia△ and South Africa based on *nifH* (401 bp) sequences with type strains of Bradyrhizobium species. The evolutionary history was inferred by using the Neighbor-joining method based on the Kimura 2-parameter model. The scale bar indicates the number of substitutions per site. The percentage of trees in which the associated taxa clustered together in the bootstrap test (1000 replicates) are shown next to the branches. Evolutionary analyses were conducted in MEGA6.

In a similar study, the soils of Botswana exhibited considerable diversity in the *Bradyrhizobium* species, (namely, *B. yuanmingense, B. daquingense* and a novel *Bradyrhizobium* sp.) that nodulated cowpea (Steenkamp et al., 2008). Studies in Senegal (West Africa) also revealed novel *Bradyrhizobium* species as the bacterial symbionts of cowpea, and these were closely related to *B. yuanmingense* (based on six loci sequence analysis) but symbiotically grouped with *B. arachidis* based on *nodC* and *nifH* gene analysis (Wade et al., 2014). A taxonomic revision of these novel *Bradyrhizobium* species showed that they belonged to *B. vignae*, which was originally isolated from cowpea nodules collected from Namibia (Figure 2, 3).

In studies of cowpea in the Okavango of Namibia, *B. pachyrhizi* was found to dominate in the acidic soils, while cowpea nodulation in the semi-humid region of Angola and Namibia was by diverse bradyrhizobial strains, most of them relating to *B. yuanmingense* and *B. daqingense*, as well as by some novel *Bradyrhizobium* species (Grönemeyer et al., 2014), which were later identified as *B. kavangense*, and *B. vignae* (Grönemeyer et al., 2015b, 2016).

Chidebe et al. (2018) recently found high molecular diversity among 122 microsymbionts isolated from Mozambican soils and characterized using BOX-PCR analysis, a finding confirmed by multilocus sequence analysis which showed that cowpea is nodulated by different *Bradyrhizobium* species (*B. elkanii, B. yuanmingense, B. diazoefficiens, B. pachyrhizi*, and novel *Bradyrhizobium* species), as well as by diverse *Rhizobium* species (*Neorhizobium galegae, Rhizobium pusense*, and *Rhizobium tropici*).

The Guinea savanna and Sudano-Sahelian regions of Ghana, as well as the low veld of South Africa were also explored for the biogeographic distribution of root-nodule bacteria nodulating cowpea in the two countries. In that study, sequence analysis of core genes (*atpD, glnII, gyrB*, and *rpoB*) and symbiotic genes (*nifH* and *nodC*) revealed the presence of highly diverse *Bradyrhizobium* species nodulating cowpea that were closely related to *B. daqingense, B. subterraneum, B. yuanmingense, B. embrapense, B. pachyrhizi*, and *B. elkanii*, as well as a number of unidentified novel *Bradyrhizobium* isolates (Mohammed et al., 2018). Multivariate analysis also showed that the distribution of these *Bradyrhizobium* species was strongly influenced by the concentration of mineral nutrients in the soils (Mohammed et al., 2018).

**FIGURE 4:**
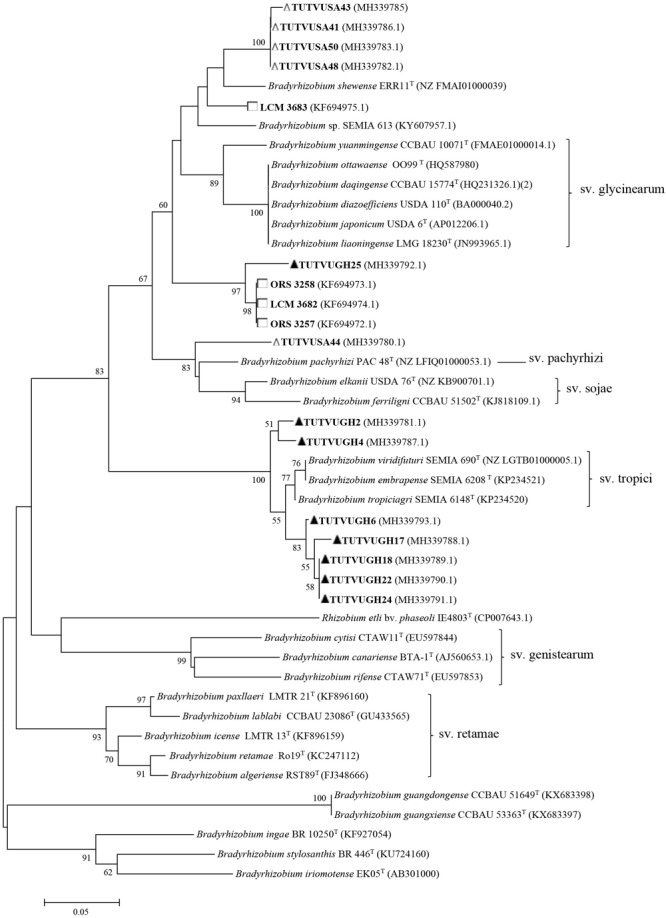
Neighbor-joining molecular phylogenetic analysis of cowpea nodulating rhizobia from ▲Ghana, □ Senegal, and △ South Africa based on *nodC* sequences with type strains of *Bradyrhizobium* species. The evolutionary history was inferred by using the Neighbor-joining method based on the Kimura 2-parameter model. The scale bar indicates the number of substitutions per site. The percentage of trees in which the associated taxa clustered together in the bootstrap test (1000 replicates) are shown next to the branches. Evolutionary analyses were conducted in MEGA6.

Some of the previously reported novel *Bradyrhizobium* sp. from Ghana, Senegal, Angola, Namibia, South Africa, and Mozambique, as well as those from our study were reanalyzed phylogenetically using previously and newly listed *Bradyrhizobium* type strains in the GenBank. The results showed that only the Senegalese isolates initially considered to be novel, were actually strains of *B. vignae*, while all the others remained novel *Bradyrhizobium* sp. with no alignment to any reference type strains in the GenBank (see Figure 2–4).

After cowpea, Bambara groundnut (*Vigna subterranea* L. Verdc.) is the second most important indigenous non-commercial food legume in Africa both in consumption and land area under cultivation. It is cultivated across the entire African continent, from Mauritania and Senegal in the West to Uganda and Tanzania in the East, and from Mali and Sudan in the North to South Africa, Zimbabwe, and Swaziland in the South. Bambara groundnut is cultivated as a sole or mixed culture (Doku, 1995), and is drought-tolerant, as well as performs well in very nutrient-poor soils due to its ability to form effective symbiosis with soil rhizobia that reduce atmospheric N_2_ to ammonia (Puozaa et al., 2017). Nitrogen contribution by Bambara groundnut under field and glasshouse conditions can vary significantly, with values ranging from 4 to 200 kg N ha^-1^ (Gueye and Bordeleau, 1988; Kumaga et al., 1994; Kishinevsky et al., 1996; Nyemba and Dakora, 2010; Mohale et al., 2013).

Analysis of nodules from Angola and Namibia have suggested that Bambara groundnut is nodulated by a diverse group of *Bradyrhizobium* species (Grönemeyer et al., 2014). Using RFLP analysis of root-nodule bacteria collected from South Africa, as well as the Guinea and sahelian Savanna of Ghana, Puozaa et al. (2017) found that diverse bradyrhizobia nodulate Bambara groundnut in those regions. These indigenous bradyrhizobia were closely related to *B. vignae* (Puozaa et al., 2017), which nodulates *Vigna subterranea, Vigna unguiculata, Arachis hypogaea*, and *Lablab purpureus* (Grönemeyer et al., 2014). However, some novel monophyletic groups were also identified which suggested the presence of novel *Bradyrhizobium* in the soils studied. Caballero-Mellado and Martinez-Romero (1996) have argued that the origins of legumes tend to coincide with the centers of diversity of their specific symbiotic bacteria. This supports the large diversity of *Bradyrhizobium* species found to nodulate Bambara groundnut in Africa (Grönemeyer et al., 2014; Puozaa et al., 2017), the center of origin of this legume. Taken together, the reports of various studies appear to suggest that *Vigna* species in Africa are largely nodulated by *Bradyrhizobium* bacteria. The huge functional diversity among microsymbionts nodulating *Vigna* indicate the potential for exploiting these diverse rhizobial populations for use as inoculants to increase cowpea and Bambara groundnut production in Africa.

## Soybean Nodulation by *Bradyrhizobium* Species

Soybean (*Glycine max* L. Merr.) belongs to the tribe *Phaseoleae*, sub-tribe *Glycininae*, and genus *Glycine*. It originated from north-eastern China and is presently cultivated worldwide under various climatic conditions (Appunu et al., 2008; Risal et al., 2010; Singh, 2010). Soybean was probably introduced into Africa by Chinese traders traveling along the east coast of Africa in the 1800s (Shurtleff and Aoyagi, 2009). The average soybean yield in Africa is 1.0 ton per hectare compared with the world average of 2.35 ton per hectare (Abate et al., 2012). Africa is one of the largest importers of soybean in the world due to low local production, (Mutegi and Zingore, 2014).

As a legume, soybean obtains about 50–60% of its N nutrition from the atmosphere (Hardarson and Atkins, 2003; Salvagiotti et al., 2008). In Africa, N contribution by soybean is about 63 kg N ha^-1^ in Malawi (van Vugt et al., 2018), and up to 290 kg N ha^-1^ in South Africa (Mapope and Dakora, 2016).

Historically, soybean was believed to be nodulated by strains of only *B. japonicum*, which are not endemic to African soils. As a result, new soybean genotypes [Tropical Glycine cross (TGx)], were bred that would nodulate freely with *Bradyrhizobium* populations indigenous to African soils (Pulver et al., 1985; Abaidoo et al., 2000). These so-called promiscuous TGx soybean genotypes can fix more N_2_ than the strict nodulating varieties from North America due to their ability to form more effective symbiosis with indigenous *Bradyrhizobium* strains in African soils (Mpepereki et al., 2000). The TGx soybean varieties also seem to have the ability to attract diverse indigenous bradyrhizobia in African soils as shown by *B. japonicum, B. diazoefficiens*, and *B. elkanii* which were recently identified as the dominant strains nodulating soybean in South Africa, Ethiopia, and Mozambique (Jaiswal et al., 2016; Naamala et al., 2016; Chibeba et al., 2017; Gyogluu et al., 2018). *B. elkanii* is also reported to be the major microsymbiont nodulating soybean in Malawian and Kenyan soils (Herrmann et al., 2014; Parr, 2014). Jaiswal et al. (2016), Naamala et al. (2016), and Gyogluu et al. (2018) have similarly found *B. elkanii* and some native *Bradyrhizobium* species to be the dominant symbionts species nodulating soybean in South Africa, Mozambique and Ethiopia. Geographically speaking, an earlier study by Abaidoo et al. (2000) also indicated that *B. elkanii* and *B. japonicum* were the most dominant bacterial symbionts isolated from root nodules of soybean in Benin, Cameroon, Ghana, Nigeria, Togo, and Uganda. Taken together, these findings from the various studies confirm the nodulation of soybean by a diverse group of bradyrhizobia, but with *B. elkanii* and *B. japonicum* being very dominant.

Poor yield of soybean when it was first introduced into new areas outside Southeast Asia, (its center of origin and domestication) was attributed to the lack of co-evolved rhizobial strains in those soils (Pulver et al., 1985; Maingi et al., 2006; Abaidoo et al., 2007; Li et al., 2010; Chianu et al., 2011; Parr, 2014; Jaiswal et al., 2016). As a result, inoculation of soybean with exotic rhizobia has been the practice when soybean is introduced into new regions. Even with the TGx soybean genotypes which can nodulate freely with indigenous rhizobia, there are instances where native strains are either ineffective or low in numbers, suggesting soybean must still be inoculated with effective strains, whether indigenous or exotic (Sanginga et al., 2000; Giller, 2001; Okogun and Sanginga, 2003; Osunde et al., 2003; Abaidoo et al., 2007; Klogo et al., 2015). Inoculation is recommended in virgin soils when there are less than 10 cells of indigenous rhizobia per gram of soil (Thies et al., 1991, 1992; Sanginga et al., 1996; Sessitsch et al., 2002; Okogun and Sanginga, 2003). A number of studies have indicated the presence of highly effective native rhizobia in African soils that can be used as inoculants for increased soybean production (Abaidoo et al., 2000, 2007; Sanginga et al., 2000; Musiyiwa et al., 2005; Tefera, 2011; Youseif et al., 2014; Klogo et al., 2015; Gyogluu et al., 2016; Chibeba et al., 2017).

## Groundnut Nodulation by Diverse Rhizobial Populations in Africa

Though native to South America, groundnut is an important grain legume in Africa (Hammons, 1982), brought by Spanish and Portuguese explorers on their voyages to Africa. Due to its ability to fix N_2_, groundnut has become a significant component of traditional cropping systems. The amount of N-fixed by groundnut can vary hugely depending on the genotype (Mokgehle et al., 2014) and the efficacy of the rhizobial strains involved in the symbiosis (Paffetti et al., 1998). In general, however, groundnut is known to have high N_2_-fixing capacity as it can obtain about 88–93% of its N nutrition from BNF and contribute up to 206 kg N ha^-1^ in cropping systems (Boddey et al., 1990; Peoples et al., 1991; Toomsan et al., 1995; Hoa et al., 2002; Osei et al., 2018). Estimates of N contribution by groundnut was up to 188 kg N ha^-1^ in South Africa (Mokgehle et al., 2014), 58–101 kg N ha^-1^ in Ghana (Konlan et al., 2013), and 19–79 kg N ha^-1^ in Zambia (Nyemba and Dakora, 2010). Despite the agronomic importance of groundnut, studies of its microsymbionts are relatively few, and cover only four African countries, namely Cameroon (Ngo Nkot et al., 2008), Morocco (El-Akhal et al., 2008), Ghana (Osei et al., 2018), and South Africa (Law et al., 2007; Steenkamp et al., 2008; Jaiswal et al., 2017).

Whether because nodule formation in groundnut is by crack entry and infection thread invasion (Tajima et al., 2008), groundnut-nodulating symbionts tend to reveal high levels of diversity and heterogeneity when obtained from different regions of Africa. This heterogeneity could be due to the high level of promiscuity reported for groundnut nodulation (Nievas et al., 2012; Jaiswal et al., 2017; Osei et al., 2018) as it can form effective symbiosis with both slow- and fast-growing bacteria belonging to the genera *Rhizobium* and *Bradyrhizobium* in African soils (Taurian et al., 2006; El-Akhal et al., 2008; de Freitas and Silva, 2013; Jaiswal et al., 2017; Osei et al., 2018). The fast-growing rhizobia that are reported to effectively nodulate groundnut in African soils, include *R. giardinii* and *R. tropici* (Taurian et al., 2006; Jaiswal et al., 2017; Osei et al., 2018).

Based on ribosomal and symbiotic gene sequence analyses, *B. yuanmingense* and *R. tropici* were identified as the microsymbionts nodulating groundnut in Ghanaian soils (Osei et al., 2018). In South Africa, however, Jaiswal et al. (2017) also reported the presence of a huge diversity of bacteria nodulating groundnut, based on ITS-RFLP, core and symbiotic gene sequence analyses, with *Rhizobium* and *Bradyrhizobium* as the major dominants of groundnut nodulation. Of the latter, *B. pachyrhizi* and novel *Bradyrhizobium* species close to *B. guangdongense* and *B. diazoefficiens* were responsible for groundnut nodulation, while of the *Rhizobium* group, *R. tropici* and other novel *Rhizobium* species also contributed to groundnut nodulation. Clearly, the taxonomic position of rhizobia nodulating groundnut is still not well defined. These results, however, do suggest that chromosomal and symbiotic genes could have the same evolutionary history.

## Nodulation of Wild Legumes by Diverse Bradyrhizobia in Africa

Besides the nodulation of economically important grain legumes, *Bradyrhizobium* species are also associated with the nodulation of wild legumes in African soils. For example, diverse photosynthetic and non-photosynthetic bradyrhizobia with distinct host-ranges have been reported in the nodulation of *Aeschynomene* species in Senegal (Nzoué et al., 2009; Molouba et al., 1999). In Senegal, effective diverse *Bradyrhizobium* sp. found in deep soil could nodulate *Acacia albida* (Dupuy and Dreyfus, 1992; Dupuy et al., 1994). The soils of the sudanean and Sahelian regions of Senegal harbor *B. elkanii and B. japonicum* as the nodulating species of the wild legumes *Pterocarpus erinaceus* and *Pterocarpus lucens* (Sylla et al., 2002). Based on amplified fragment length polymorphism (AFLP), 16S–23S rRNA, SDS-PAGE and 16S rRNA-RFLP, diverse *Bradyrhizobium* sp. nodulating various wild legume genera (*Abrus, Alysicarpus, Bryaspis, Chamaecrista, Cassia, Crotalaria, Desmodium, Eriosema, Indigofera, Moghania, Rhynchosia, Sesbania, Tephrosia*, and *Zornia*) have been identified in the arid regions of Senegal (West Africa) (Doignon-Bourcier et al., 1999, 2000). Furthermore, microsymbionts isolated from *Zornia glochidiata* in the degraded semi-arid soils of the Sahelian ecosystem were identified as *B. liaoningense, B. yuanmingense* and *B. japonicum* based on 16S–23S rRNA and *recA* gene sequence analysis (Gueye et al., 2009).

The Cape Floristic region is largely semi-arid with nutrient-poor, acidic soils (Witkowski and Mitchell, 1987; Manning and Goldblatt, 2012) that is home to many endemic papilionoid tribes such as Crotalarieae, Podalyrieae, Psoraleeae, and Indigofereae (Goldblatt and Manning, 2002; Linder, 2003; Manning and Goldblatt, 2012), which play an important ecological role in providing symbiotic N to the ecosystem (Sprent, 2009; Sprent et al., 2010, 2013). Interestingly, the nodulation of some fynbos legumes is by *Bradyrhizobium* species. Recently, Lemaire et al. (2015) identified diverse populations of *Bradyrhizobium* as the microsymbionts of Cape legumes. *B. canariense*, which is highly acid-tolerant (Vinuesa et al., 2005), was isolated from *Indigofera gracilis*, and *B. elkanii* from *Indigofera frutescens* and *Tephrosia capensis* in the Cape fynbos of South Africa.

*Bradyrhizobium* nodulation has been reported for *Argyrolobium rupestre* and *Argyrolobium sericeum* from the Genisteae tribe, *Leobordea pulchra, L*. *divaricate, L*. *lanceolate*, and *Pearsonia obovate* from the Crotalarieae tribe, as well as *Chamaecrista* from the Cassieae tribe (Beukes et al., 2016), which have their centers of divergence in Southern Africa (Polhill and VanWyk, 2005). However, this diverse group of *Bradyrhizobium* were closely related to six novel species without alignment to known reference strains. This suggests the widespread nodulation of legumes in Africa by *Bradyrhizobium* species.

Furthermore, a phylogenetically diverse group of *Bradyrhizobium* genospecies were also found to be the microsymbionts nodulating *Crotalaria, Indigofera*, and *Erythrina brucei* in Ethiopia (Aserse et al., 2012). In another study, diverse *Bradyrhizobium* species were the natural microsymbionts of the tree legumes *Acacia saligna, Faidherbia albida, Erythrina brucei, Millettia ferruginea*, and *Albizia gummifera* sampled from dry, hot, semi-arid to moist cold contrasting environments in Ethiopia (Wolde-meskel et al., 2004). In an earlier report, *Faidherbia albida* was the most frequently used host for trapping *Bradyrhizobium* in Africa (Odee et al., 2002). Multilocus sequence analysis later confirmed that *B. huanghuaihaiense, B. yuanmingense, B. pachyrhizi* and some novel *Bradyrhizobium* species were the natural bacterial symbionts of *Faidherbia albida, Acacia saligna, Erythrina brucei, Albizia gummifera*, and *Millettia ferruginea* in Ethiopian soils (Degefu et al., 2017). Analysis of chromosomal (*glnII, recA, gyrB*, and *dnaK*) and symbiotic (*nodA, nodC, nifD*, and *nifH*) genes of *Bradyrhizobium* obtained from wild African legumes suggested their separate evolutionary history, and that the symbiotic genes were likely to be of African origin (Beukes et al., 2016; Degefu et al., 2017).

A diverse group of *Bradyrhizobium* species close to *B. canariense, B. cytisi, B. rifense, B. japonicum*, and *B. elkanii* were found to nodulate *Lupinus micranthus* in Africa, however, multilocus sequence analysis and *nodC* phylogeny defined them as symbiovar genistearum (Bourebaba et al., 2016). In Algeria, *B. algeriense* could also nodulate *Retama raetam, Lupinus micranthus, Lupinus albus*, and *Genista numidica*, but not *Lupinus angustifolius* or *Glycine max* (Ahnia et al., 2018). In an earlier report, *B. canariense* isolated in Spain showed the ability to nodulate serradella and *Lupinus cosentinii* in South Africa (Stȩpkowski et al., 2005).

## Factors Influencing *Bradyrhizobium* Distribution in African Soils

Many factors are known to influence bacterial distribution in soils, which include the presence of legume host, and environmental conditions such as soil factors (Keyser and Li, 1992; Wani et al., 1995; Dakora and Keya, 1997; Paffetti et al., 1998; Zahran, 1999; Peoples et al., 2009; Abi-Ghanem et al., 2013; Vitousek et al., 2013; Puozaa et al., 2017). These factors can affect all aspects of legume nodulation and N_2_ fixation which include a decrease in rhizobial survival and diversity in soils as well as their direct effect on nitrogenase activity (Serraj, 2004). Native rhizobia and bradyrhizobia easily develop adaptive mechanisms for surviving stress (Dakora, 2012), with the latter generally exhibiting better resistance than the former. So far, 52 *Bradyrhizobium* symbionts have been identified globally for their ability to nodulate the legume plants (Table 1). Of these, only ten (*B. algerianse, B. cytisi, B. kavangense, B. namibiense, B. subterraneum, B. retamae, B. rifense, B. ripae, B. vignae*, and *B. shewense*) have an African origin with an ability to tolerate acidic to alkaline conditions (pH 4–9) (Chahboune et al., 2011; Chahboune et al., 2012; Guerrouj et al., 2013; Grönemeyer et al., 2015a,b, 2016, 2017; Ahnia et al., 2018; Bünger et al., 2018) (see Table 1). It therefore appears that the physico-chemical properties of soils play an important role in influencing the diversity of bradyrhizobial community in a niche (Jaiswal et al., 2016; Ojha et al., 2017; Puozaa et al., 2017; Rathi et al., 2018).

Rhizobial strains often perform poorly when introduced into new habitats, thus implying that their symbiotic effectiveness is often adapted to environmental factors of their local niche such as soil temperature (Roughley, 1970; Zhang et al., 2003; Suzuki et al., 2014), soil pH (Botha et al., 2004; Temprano-Vera et al., 2018), soil texture (Law et al., 2007), and host-plant type (Pule-Meulenberg et al., 2010; Jaiswal et al., 2016;Puozaa et al., 2017).

Low soil pH is for example an abiotic stress that can affect growth of the legume host, the microsymbiont, and their interaction due to high concentrations of protons that have direct or indirect effects such as forming toxic trivalent ions with aluminum, and/or reducing the availability of nutritionally and symbiotically important minerals such as Ca, Mg, P, and Mo (Indrasumunar et al., 2011; Dakora, 2012; Jaiswal et al., 2018). A recent study has shown that the occurrence and abundance of diverse cowpea rhizobial populations in Kenya was influenced by soil pH (Ndungu et al., 2018), which typically affects the availability of endogenous mineral nutrients in soils.

It is believed that the host plays a dominant role in shaping the choice of bacterial symbionts for the symbiosis (Hollowell et al., 2016). Consistent with this argument, Puozaa et al. (2017) found that when seeds with different seed coat pigmentations were planted in the same hole, they attracted different bradyrhizobial genotypes. This clearly supports the view that each host plant selects specific effective bacterial symbionts over those that are less effective for its nodulation. If so, it remains to be assessed why highly effective introduced strains fail to outcompete less effective native rhizobia.

Furthermore, the results of Puozaa et al. (2017) showed that Bambara groundnut nodulation was associated with much highly diverse bradyrhizobial populations in the drier Morwe region of South Africa (where rainfall was very low) when compared to the moist Ghanaian soils. These results are consistent with the finding that more diverse bradyrhizobial populations were found in soils from the drier or less-humid environments of Africa relative to wetter regions (Law et al., 2007; Krasova-Wade et al., 2003; Grönemeyer et al., 2014).

Besides soil moisture, Mohammed et al. (2018) also reported recently that soil mineral nutrients can influence the distribution of bradyrhizobia nodulating cowpea in the soils of South Africa and Ghana. Here, the South African bacterial symbionts were highly influenced by the endogenous soil concentrations of N, P, and Na, in contrast to Ghana, where B, Mn, and Fe had a major influence in the distribution of soil bradyrhizobia.

## Current Challenges and Future Directions of Legume Root Nodulation in African Soils

The data collected here have strongly contributed to the growing body of knowledge on the size and efficacy of *Bradyrhizobium* populations in African soils, as well as our understanding of the evolution of this important group of microsymbionts. Africa could be a hotspot of bradyrhizobial biodiversity, a view that compels us to search for more novel bradyrhizobia in African soils in order to learn more about their biogeography. The presence of *B. yuanmingense* and *B. elkanii* in almost all the African soils studied probably suggest that strains of these species may be the most widely distributed in African soils.

Whether bradyrhizobial distribution in Africa suggests sympatric or allopatric speciation, remains to be assessed. However, it is interesting that many highly specialized endemic novel *Bradyrhizobium* species have been identified, while others present in the same niche, microclimate, and edaphic conditions, and suspected to be novel species remain to be delineated through systematic studies. That the rhizosphere environment and the host plant play a major role in the selection of the bacterial symbiont probably explains why only highly adapted indigenous bradyrhizobia often overcome introduced inoculant strains for root nodulation in legumes. So far, very few countries have been explored for the biogeography of *Bradyrhizobium* in African soils. Studies of different regions for new rhizobia and legume germplasm could lead to the discovery of novel microsymbionts that would support agricultural productivity in the continent.

## Author Contributions

SJ collected the references and wrote this manuscript. FD critically reviewed and revised the article.

## Conflict of Interest Statement

The authors declare that the research was conducted in the absence of any commercial or financial relationships that could be construed as a potential conflict of interest.

## References

[B1] Abaidoo R. C. Keyser H. H. Singleton P. W. Borthakur D. (2000). *Bradyrhizobium* spp.(TGx) isolates nodulating the new soybean cultivars in Africa are diverse and distinct from bradyrhizobia that nodulate North American soybeans. *Int. J. Syst. Evol. Microbiol.* 50 225–234. 10.1099/00207713-50-1-225 10826808

[B2] Abaidoo R. C. Keyser H. H. Singleton P. W. Dashiell K. E. Sanginga N. (2007). Population size, distribution, and symbiotic characteristics of indigenous *Bradyrhizobium* spp. that nodulate TGx soybean genotypes in Africa. *Appl. Soil Ecol.* 35 57–67. 10.1016/j.apsoil.2006.05.006

[B3] Abate T. Alene A. D. Bergvinson D. Shiferaw B. Silim S. Orr A. (2012). *Tropical Grain Legumes in Africa and South Asia: knowledge and Opportunities.* Patancheru: International Crops Research Institute for the Semi-Arid Tropics.

[B4] Abi-Ghanem R. Bodah E. Wood M. Braunwart K. (2013). Potential breeding for high nitrogen fixation in *Pisum sativum* L: germplasm phenotypic characterization and genetic investigation. *Am. J. Plant Sci.* 4:1597 10.4236/ajps.2013.48193

[B5] Ahnia H. Bourebaba Y. Durán D. Boulila F. Palacios J. M. Rey L. (2018). *Bradyrhizobium algeriense* sp. nov., a novel species isolated from effective nodules of *Retama sphaerocarpa* from Northeastern Algeria. *Syst. Appl. Microbiol.* 41 333–339. 10.1016/j.syapm.2018.03.004 29656850

[B6] Akibode C. S. (2011). *Trends in the Production, Trade, and Consumption of Food Legume Crops in Sub-Saharan Africa.* M. Sc. thesis, Michigan State University East Lansing, MI.

[B7] Appunu C. Angele N. Laguerre G. (2008). Genetic diversity of native bradyrhizobia isolated from soybeans (*Glycine max* L.) in different agricultural-ecological-climatic regions of India. *Appl. Environ. Microbiol.* 74 5991–5996. 10.1128/AEM.01320-08 18676699 PMC2565974

[B8] Araújo J. Flores-Félix J. D. Igual J. M. Peix A. González-Andrés F. Díaz-Alcántara C. A. (2017). *Bradyrhizobium cajani* sp. nov. isolated from nodules of *Cajanus cajan*. *Int. J. Syst. Evol. Microbiol.* 67 2236–2241. 10.1099/ijsem.0.001932 28671523

[B9] Aserse A. A. Rasanen L. A. Aseffa F. Hailemariam A. Lindstrom K. (2012). Phylogenetically diverse groups of *Bradyrhizobium* isolated from nodules of *Crotalaria* spp., *Indigofera* spp., *Erythrina brucei* and *Glycine max* growing in Ethiopia. *Mol. Phylogenet. Evol.* 65 595–609. 10.1016/j.ympev.2012.07.008 22842091

[B10] Aserse A. A. Woyke T. Kyrpides N. C. Whitman W. B. Lindström K. (2017). Draft genome sequences of *Bradyrhizobium shewense* sp. nov. ERR11T and *Bradyrhizobium yuanmingense* CCBAU 10071T. *Stand. Genomic Sci.* 12:74. 10.1186/s40793-017-0283-x 29225730 PMC5717998

[B11] Aticho A. Elias E. Diels J. (2011). Comparative analysis of soil nutrient balance at farm level: a case study in Jimma Zone, Ethiopia. *Int. J. Soil Sci.* 6 259–266. 10.3923/ijss.2011.259.266

[B12] Belane A. K. Dakora F. D. (2010). Symbiotic N2 fixation in 30 field-grown cowpea (*Vigna unguiculata* L. Walp.) genotypes in the Upper West Region of Ghana measured using 15N natural abundance. *Biol. Fertil. Soils* 46 191–198. 10.1007/s00374-009-0415-6

[B13] Beukes C. W. Stepkowski T. Venter S. N. Cłapa T. Phalane F. L. le Roux M. M. (2016). Crotalarieae and Genisteae of the South African Great Escarpment are nodulated by novel *Bradyrhizobium* species with unique and diverse symbiotic loci. *Mol. Phylogenet. Evol.* 100 206–218. 10.1016/j.ympev.2016.04.011 27068839

[B14] Beukes C. W. Venter S. N. Law I. J. Phalane F. L. Steenkamp E. T. (2013). South African papilionoid legumes are nodulated by diverse *Burkholderia* with unique nodulation and nitrogen-fixation loci. *PLoS One* 8:e68406. 10.1371/journal.pone.0068406 23874611 PMC3708930

[B15] Bezner Kerr R. Snapp S. Chirwa M. Shumba L. Msachi R. (2007). Participatory research on legume diversification with Malawian smallholder farmers for improved human nutrition and soil fertility. *Exp. Agric.* 43 437–453. 10.1017/S0014479707005339

[B16] Boddey R. M. Urquiaga S. Neves M. C. P. Peres J. (1990). Quantification of the contribution of N2 fixation to field-grown grain legumes—a strategy for the practical application of the 15N isotope dilution technique. *Soil Biol. Biochem.* 22 649–655. 10.1016/0038-0717(90)90011-N

[B17] Botha W. J. Jaftha J. B. Bloem J. F. Habig J. H. Law I. J. (2004). Effect of soil bradyrhizobia on the success of soybean inoculant strain CB 1809. *Microbiol. Res.* 159 219–231. 10.1016/j.micres.2004.04.004 15462522

[B18] Bourebaba Y. Durán D. Boulila F. Ahnia H. Boulila A. Temprano F. (2016). Diversity of *Bradyrhizobium* strains nodulating *Lupinus micranthus* on both sides of the Western Mediterranean: Algeria and Spain. *Syst. Appl. Microbiol.* 39 266–274. 10.1016/j.syapm.2016.04.006 27236566

[B19] Bünger W. Grönemeyer J. L. Sarkar A. Reinhold-Hurek B. (2018). *Bradyrhizobium ripae* sp. nov., a nitrogen-fixing symbiont isolated from nodules of wild legumes in Namibia. *Int. J. Syst. Evol. Microbiol.* 68 3688–3695. 10.1099/ijsem.0.002955 30247121

[B20] Caballero-Mellado J. Martinez-Romero E. (1996). Rhizobium phylogenies and bacterial genetic diversity. *Crit. Rev. Plant Sci.* 15 113–140. 10.1080/07352689.1996.10393183

[B21] Chahboune R. Carro L. Peix A. Barrijal S. Velázquez E. Bedmar E. J. (2011). *Bradyrhizobium cytisi* sp. nov. isolated from effective nodules of *Cytisus villosusin* Morocco. *Int. J. Syst. Evol. Microbiol.* 61 2922–2927. 10.1099/ijs.0.027649-0 21257682

[B22] Chahboune R. Carro L. Peix A. Ramírez-Bahena M. H. Barrijal S. (2012). *Bradyrhizobium rifense* sp. nov. isolated from effective nodules of *Cytisus villosus* grown in the Moroccan Rif. *Syst. Appl. Microbiol.* 35 302–305. 10.1016/j.syapm.2012.06.001 22795906

[B23] Chang Y. L. Wang J. Y. Wang E. T. Liu H. C. Sui X. H. Chen W. X. (2011). *Bradyrhizobium lablabi* sp. nov., isolated from effective nodules of *Lablab purpureus* and *Arachis hypogaea*. *Int. J. Syst. Evol. Microbiol.* 61 2496–2502. 10.1099/ijs.0.027110-0 21112989

[B24] Chianu J. N. Nkonya E. M. Mairura F. S. Chianu J. N. Akinnifesi F. K. (2011). Biological nitrogen fixation and socioeconomic factors for legume production in sub-Saharan Africa: a review. *Agron. Sustain. Develop.* 31 139–154. 10.1098/rstb.2012.0284 24535391 PMC3928888

[B25] Chibeba A. M. Kyei-Boahen S. de Fátima Guimarães M. Nogueira M. A. Hungria M. (2017). Isolation, characterization and selection of indigenous *Bradyrhizobium* strains with outstanding symbiotic performance to increase soybean yields in Mozambique. *Agri. Ecosyst. Environ.* 246 291–305. 10.1016/j.agee.2017.06.017 28775390 PMC5521954

[B26] Chidebe I. N. Jaiswal S. K. Dakora F. D. (2018). Distribution and phylogeny of microsymbionts associated with cowpea (*Vigna unguiculata*) nodulation in three agroecological regions of Mozambique. *Appl. Environ. Microbiol.* 84 1–25. 10.1128/AEM.01712-17 29101189 PMC5752868

[B27] Costa E. M. Nóbrega R. S. A. Carvalho F. Trochmann A. Ferreira L. V. M. Moreira F. M. S. (2013). Plant growth promotion and genetic diversity of bacteria isolated from cowpea nodules. *Pesq. Agropec. Bras.* 48 1275–1284. 10.1590/S0100-204X2013000900012

[B28] Crews T. E. Peoples M. B. (2004). Legume versus fertilizer sources of nitrogen: ecological trade-offs and human needs. *Agri. Ecosyst. Environ.* 102 279–297. 10.1016/j.agee.2003.09.018

[B29] da Costa E. M. Guimarães A. A. de Carvalho T. S. Rodrigues T. L. de Almeida Ribeiro P. R. Lebbe L. (2018). *Bradyrhizobium forestalis* sp. nov., an efficient nitrogen-fixing bacterium isolated from nodules of forest legume species in the Amazon. *Arch. Microbiol.* 200 743–752. 10.1007/s00203-018-1486-2 29396618

[B30] da Costa E. M. Guimarães A. A. Vicentin R. P. de Almeida Ribeiro P. R. Leão A. C. R. Balsanelli E. (2017). *Bradyrhizobium brasilense* sp. nov., a symbiotic nitrogen-fixing bacterium isolated from Brazilian tropical soils. *Arch. Microbiol.* 199 1211–1221. 10.1007/s00203-017-1390-1 28551732

[B31] da Silva K. De Meyer S. E. Rouws L. F. Farias E. N. dos Santos M. A. O’Hara G. (2014). *Bradyrhizobium ingae* sp. nov., isolated from effective nodules of *Inga laurina* grown in Cerrado soil. *Int. J. Syst. Evol. Microbiol.* 64 3395–3401. 10.1099/ijs.0.063727-0 25013231

[B32] Dakora F. D. (2012). Root-nodule bacteria isolated from native *Amphithalea ericifolia* and four indigenous *Aspalathus* species from the acidic soils of the South African fynbos are tolerant to very low pH. *Afr. J. Biotechnol.* 11 3766–3772.

[B33] Dakora F. D. Keya S. O. (1997). Contribution of legume nitrogen fixation to sustainable agriculture in Sub-Saharan Africa. *Soil Biol. Biochem.* 29 809–817. 10.1016/S0038-0717(96)00225-8

[B34] de Freitas A. D. S. Silva T. A. (2013). Phenotypic and molecular characteristics of rhizobia isolated from nodules of peanut (*Arachis hypogaea* L.) grown in Brazilian Spodosols. *Afr. J. Biotechnol.* 12:2147 10.5897/AJB11.1574

[B35] de Matos G. F. Zilli J. E. de Araújo J. L. S. Parma M. M. Melo I. S. Radl V. (2017). *Bradyrhizobium sacchari* sp. nov., a legume nodulating bacterium isolated from sugarcane roots. *Arch. Microbiol.* 199 1251–1258. 10.1007/s00203-017-1398-6 28601967

[B36] Degefu T. Wolde-meskel E. Woliy K. Frostegård Å (2017). Phylogenetically diverse groups of *Bradyrhizobium* isolated from nodules of tree and annual legume species growing in Ethiopia. *Syst. Appl. Microbiol.* 40 205–214. 10.1016/j.syapm.2017.04.001 28499469

[B37] Delamuta J. R. M. Ribeiro R. A. Araújo J. L. S. Rouws L. F. M. Zilli J. É Parma M. M. (2016). *Bradyrhizobium stylosanthis* sp. nov., comprising nitrogen-fixing symbionts isolated from nodules of the tropical forage legume *Stylosanthes* spp. *IJSEM* 66 3078–3087. 10.1099/ijsem.0.001148 27169861

[B38] Delamuta J. R. M. Ribeiro R. A. Ormeno-Orrillo E. Melo I. S. Martínez-Romero E. Hungria M. (2013). Polyphasic evidence supporting the reclassification of *Bradyrhizobium japonicum* group Ia strains as *Bradyrhizobium diazoefficiens* sp. nov. *Int. J. Syst. Evol. Microbiol.* 63 3342–3351. 10.1099/ijs.0.049130-0 23504968

[B39] Delamuta J. R. M. Ribeiro R. A. Ormeño-Orrillo E. Parma M. M. Melo I. S. Martínez-Romero E. (2015). *Bradyrhizobium tropiciagri* sp. nov. and *Bradyrhizobium embrapense* sp. nov., nitrogen-fixing symbionts of tropical forage legumes. *IJSEM* 65 4424–4433. 10.1099/ijsem.0.000592 26362866

[B40] Doignon-Bourcier F. Sy A. Willems A. Torck U. Dreyfus B. Gillis M. (1999). Diversity of bradyrhizobia from 27 tropical *Leguminosae* species native of Senegal. *Syst. Appl. Microbiol.* 22 647–661. 10.1016/S0723-2020(99)80018-6 10794153

[B41] Doignon-Bourcier F. Willems A. Coopman R. Laguerre G. Gillis M. de Lajudie P. (2000). Genotypic characterization of *Bradyrhizobium* strains nodulating small Senegalese legumes by 16S-23S rRNA intergenic gene spacers and amplified fragment length polymorphism fingerprint analyses. *Appl. Environ. Microbiol.* 66 3987–3997. 10.1128/AEM.66.9.3987-3997.2000 10966419 PMC92249

[B42] Doku E. V. (1995). “Country report on Ghana,” in *Conservation and Improvement of Bambara Groundnut (Vigna subterranea (L.) Verdc* eds Heller B. Mushonga J. (Harare: Department of Research & Specialist Services).

[B43] Dupuy N. Willems A. Pot B. Dewettinck D. Vandenbruaene I. Maestrojuan G. (1994). Phenotypic and genotypic characterization of bradyrhizobia nodulating the leguminous tree *Acacia albida*. *IJSEM* 44 461–473. 10.1099/00207713-44-3-461 7520737

[B44] Dupuy N. C. Dreyfus B. L. (1992). *Bradyrhizobium* populations occur in deep soil under the leguminous tree *Acacia albida*. *Appl. Environ. Microbiol.* 58 2415–2419. 16348745 10.1128/aem.58.8.2415-2419.1992 PMC195796

[B45] Durán D. Rey L. Mayo J. Zúñiga-Dávila D. Imperial J. Ruiz-Argüeso T. (2014a). *Bradyrhizobium paxllaeri* sp. nov. and *Bradyrhizobium icense* sp. nov., nitrogen-fixing rhizobial symbionts of Lima bean (*Phaseolus lunatus* L.) in Peru. *IJSEM* 64 2072–2078. 10.1099/ijs.0.060426-0 24664579

[B46] Durán D. Rey L. Navarro A. Busquets A. Imperial J. Ruiz-Argüeso T. (2014b). *Bradyrhizobium valentinum* sp. nov., isolated from effective nodules of Lupinus mariae-josephae, a lupine endemic of basic-lime soils in Eastern Spain. *Syst. Appl. Microbiol.* 37 336–341. 10.1016/j.syapm.2014.05.002 24958607

[B47] El-Akhal M. R. Rincón A. Arenal F. Lucas M. M. El Mourabit N. Barrijal S. (2008). Genetic diversity and symbiotic efficiency of rhizobial isolates obtained from nodules of *Arachis hypogaea* in north western Morocco. *Soil Biol. Biochem.* 40 2911–2914. 10.1016/j.soilbio.2008.08.005

[B48] Evans J. R. von Caemmerer S. (2011). Enhancing photosynthesis. *Plant Physiol.* 155:19. 10.1104/pp.110.900402 21205631 PMC3075791

[B49] Giller K. E. (2001). *Nitrogen Fixation in Tropical Cropping Systems.* Wallingford, CT: CABI Publishing 10.1079/9780851994178.0000

[B50] Goldblatt P. Manning J. C. (2002). Plant diversity of the Cape region of southern Africa. *Ann. Mol. Bot. Gard.* 89 281–302. 10.2307/3298566

[B51] Grönemeyer J. L. Bünger W. Reinhold-Hurek B. (2017). *Bradyrhizobium namibiense* sp. nov., a symbiotic nitrogen-fixing bacterium from root nodules of *Lablab purpureus*, hyacinth bean, in Namibia. *IJSEM* 67 4884–4891. 10.1099/ijsem.0.002039 29034855

[B52] Grönemeyer J. L. Chimwamurombe P. Reinhold-Hurek B. (2015a). *Bradyrhizobium subterraneum* sp. nov., a symbiotic nitrogen-fixing bacterium from root nodules of groundnuts. *Int. J. Syst. Evol. Microbiol.* 65 3241–3247. 10.1099/ijsem.0.000403 26198108

[B53] Grönemeyer J. L. Hurek T. Reinhold-Hurek B. (2015b). *Bradyrhizobium kavangense* sp. nov., a symbiotic nitrogen-fixing bacterium from root nodules of traditional Namibian pulses. *Int. J. Syst. Evol. Microbiol.* 65 4886–4894. 10.1099/ijsem.0.000666 26446190

[B54] Grönemeyer J. L. Hurek T. Bünger W. Reinhold-Hurek B. (2016). *Bradyrhizobium vignae* sp. nov., a nitrogen-fixing symbiont isolated from effective nodules of Vigna and Arachis. *Int. J. Syst. Evol. Microbiol.* 66 62–69. 10.1099/ijsem.0.000674 26463703

[B55] Grönemeyer J. L. Kulkarni A. Berkelmann D. Hurek T. Reinhold-Hurek B. (2014). Rhizobia indigenous to the Okavango region in Sub-Saharan Africa: diversity, adaptations, and host specificity. *Appl. Environ. Microbiol.* 80 7244–7257. 10.1128/AEM.02417-14 25239908 PMC4249195

[B56] Guerrouj K. Ruíz-Díez B. Chahboune R. Ramírez-Bahena M. H. Abdel-moumen H. Quiñones M. A. (2013). Definition of a novel symbiovar (sv. retamae) within *Bradyrhizobium retamae* sp. nov., nodulating *Retama sphaerocarpa* and *Retama monosperma*. *Syst. Appl. Microbiol.* 36 218–223. 10.1016/j.syapm.2013.03.001 23602626

[B57] Gueye F. Moulin L. Sylla S. Ndoye I. Béna G. (2009). Genetic diversity and distribution of *Bradyrhizobium* and *Azorhizobium* strains associated with the herb legume *Zornia glochidiata* sampled from across Senegal. *Syst. Appl. Microbiol.* 32 387–399. 10.1016/j.syapm.2009.04.004 19493641

[B58] Gueye M. Bordeleau L. M. (1988). Nitrogen fixation in *Bambara groundnut, Voandzeia subterranea* (L.) Thouars. Mircen. *J. Appl. Microbiol. Biotechnol.* 4 365–375. 10.1007/BF01096142

[B59] Guimarães A. A. Jaramillo P. M. D. Nóbrega R. S. A. Florentino L. A. Silva K. B. Moreira F. M. S. (2012). Genetic and symbiotic diversity of nitrogen-fixing bacteria isolated from agricultural soils in the western Amazon by using cowpea as the trap plant. *Appl. Environ. Microbiol.* 78 6726–6733. 10.1128/AEM.01303-12 22798370 PMC3426679

[B60] Gyogluu C. Boahen S. K. Dakora F. D. (2016). Response of promiscuous-nodulating soybean (*Glycine max* L. Merr.) genotypes to *Bradyrhizobium* inoculation at three field sites in Mozambique. *Symbiosis* 69 81–88. 10.1007/s13199-015-0376-5

[B61] Gyogluu C. Mohammed M. Jaiswal S. K. Kyei-Boahen S. Dakora F. D. (2018). Assessing host range, symbiotic effectiveness, and photosynthetic rates induced by native soybean rhizobia isolated from Mozambican and South African soils. *Symbiosis* 75 257–266. 10.1007/s13199-017-0520-5 29997418 PMC6015603

[B62] Hammons R. O. (1982). “Origin and early history of peanut,” in *Peanut Science and Technology* eds Pattee H. E. Young C. T. (Yoakum, TX: American Peanut Research and Education Society) 1–20.

[B63] Hardarson G. Atkins C. (2003). Optimising biological N2 fixation by legumes in farming systems. *Plant soil* 252 41–54. 10.1023/A:1024103818971

[B64] Helene L. C. F. Delamuta J. R. M. Ribeiro R. A. Hungria M. (2017). *Bradyrhizobium mercantei* sp. nov., a nitrogen-fixing symbiont isolated from nodules of *Deguelia costata* (syn. *Lonchocarpus costatus*). *IJSEM* 67 1827–1834. 10.1099/ijsem.0.001870 28639930

[B65] Helene L. C. F. Delamuta J. R. M. Ribeiro R. A. Ormeño-Orrillo E. Rogel M. A. Martínez-Romero E. (2015). *Bradyrhizobium viridifuturi* sp. nov., encompassing nitrogen-fixing symbionts of legumes used for green manure and environmental services. *IJSEM* 65 4441–4448. 10.1099/ijsem.0.000591 26362781

[B66] Herrmann L. Chotte J. L. Thuita M. Lesueur D. (2014). Effects of cropping systems, maize residues application and N fertilization on promiscuous soybean yields and diversity of native rhizobia in Central Kenya. *Pedobiology* 57 75–85. 10.1016/j.pedobi.2013.12.004

[B67] Hoa N. T. L. Thao T. Y. Lieu P. Herridge D. F. (2002). “N2 fixation of groundnut in the eastern region of south Vietnam,” in *Inoculants and Nitrogen Fixation of Legumes in Vietnam* ed. Herridge D. F. (Canberra: ACIAR) 19–28.

[B68] Hollowell A. C. Regus J. U. Turissini D. Gano-Cohen K. A. Bantay R. Bernardo A. (2016). Metapopulation dominance and genomic-island acquisition of *Bradyrhizobium* with superior catabolic capabilities. *Proc. R. Soc. B* 283:20160496. 10.1098/rspb.2016.0496 27122562 PMC4855393

[B69] Hungria M. Vargas M. A. (2000). Environmental factors affecting N2 fixation in grain legumes in the tropics, with an emphasis on Brazil. *Field Crops Res.* 65 151–164. 10.1016/S0378-4290(99)00084-2

[B70] Indrasumunar A. Dart P. J. Menzies N. W. (2011). Symbiotic effectiveness of *Bradyrhizobium japonicum* in acid soils can be predicted from their sensitivity to acid soil stress factors in acidic agar media. *Soil Biol. Biochem.* 43 2046–2052. 10.1016/j.soilbio.2011.05.022

[B71] Islam M. S. Kawasaki H. Muramatsu Y. Nakagawa Y. Seki T. (2008). *Bradyrhizobium iriomotense* sp. nov., isolated from a tumor-like root of the legume *Entada koshunensis* from Iriomote Island in Japan. *Biosci. Biotech. Biochem.* 72 1416–1429. 10.1271/bbb.70739 18540091

[B72] Jaiswal S. K. Beyan S. M. Dakora F. D. (2016). Distribution, diversity and population composition of soybean-nodulating bradyrhizobia from different agro-climatic regions in Ethiopia. *Biol. Fertil. Soils* 52 725–738. 10.1007/s00374-016-1108-6

[B73] Jaiswal S. K. Msimbira L. A. Dakora F. D. (2017). Phylogenetically diverse group of native bacterial symbionts isolated from root nodules of groundnut (*Arachis hypogaea* L.) in South Africa. *Syst. Appl. Microbiol.* 40 215–226. 10.1016/j.syapm.2017.02.002 28372899 PMC5460907

[B74] Jaiswal S. K. Naamala J. Dakora F. D. (2018). Nature and mechanisms of aluminium toxicity, tolerance and amelioration in symbiotic legumes and rhizobia. *Biol. Fertil. Soils* 54 309–318. 10.1007/s00374-018-1262-0 PMC6560468 31258230

[B75] Jaramillo P. M. D. Guimarães A. A. Florentino L. A. Silva K. B. Nóbrega R. S. A. Moreira F. M. S. (2013). Symbiotic nitrogen-fixing bacterial populations trapped from soils under agroforestry systems. *Sci. Agric.* 70 397–404. 10.1590/S0103-90162013000600004

[B76] Jordan D. C. (1982). Transfer of *Rhizobium japonicum* Buchanan 1980 to *Bradyrhizobium* gen. nov., a genus of slow-growing, root nodule bacteria from leguminous plants. *Int. J. Syst. Evol. Microbiol.* 32 136–139. 10.1099/00207713-32-1-136

[B77] Kessy J. F. Nsokko E. Kaswamila A. Kimaro F. (2016). Analysis of drivers and agents of deforestation and forest degradation in Masito forests, Kigoma, Tanzania. *Int. J. Asian Soc. Sci.* 6 93–107. 10.18488/journal.1/2016.6.2/1.2.93.107

[B78] Keyser H. H. Li F. (1992). “Potential for increasing biological nitrogen fixation in soybean,” in *Biological Nitrogen Fixation for Sustainable Agriculture* eds Ladha J. K. George T. Bohlool C. (Berlin: Springer) 119–135. 10.1007/978-94-017-0910-1_7

[B79] Kishinevsky B. D. Zur M. Friedman Y. Meromi G. Ben-Moshe E. Nemas C. (1996). Variation in nitrogen fixation and yield in landraces of *Bambara groundnut* (*Vigna subterranea* L.). *Field Crops Res.* 48 57–64. 10.1016/0378-4290(96)00037-8

[B80] Klogo P. Ofori J. K. Amaglo H. (2015). Soybean (*Glycine max* (L) Merill) promiscuity reaction to indigenous bradyrhizobia inoculation in some Ghanaian soils. *Int. J. Sci. Tech. Res.* 4 306–313.

[B81] Konlan S. Sarkodies-Addo J. Asare E. Kombiok J. M. (2013). Groundnut (*Arachis hypogaea* L.) varietal response to spacing in the Guinea Savanna agro-ecological zone of Ghana: nodulation and nitrogen fixation. *Agric. Biol. J. North Am.* 4 324–335. 10.5251/abjna.2013.4.3.324.335

[B82] Krasova-Wade T. Ndoye I. Braconnier S. Sarr B. De Lajudie P. Neyra M. (2003). Diversity of indigeneous bradyrhizobia associated with three cowpea cultivars (*Vigna unguiculata* (L.) Walp.) grown under limited and favorable water conditions in Senegal (West Africa). *Afr. J. Biotechnol.* 2 13–22. 10.5897/AJB2003.000-1003

[B83] Kumaga F. Danso S. K. Zapata F. (1994). Time-course of nitrogen fixation in two *Bambara groundnut* (*Vigna subterranea* L. Verdc.) cultivars. *Biol. Fertil. Soils* 18 231–236. 10.1007/BF00647672

[B84] Kuykendall L. M. Saxena B. Devine T. E. Udell S. E. (1992). Genetic diversityin *Bradyrhizobium japonicum* Jordan, 1982 and aproposal for *Bradyrhizobium elkanii* sp. nov. *Can. J. Microbiol.* 38 501–505. 10.1139/m92-082

[B85] Lal R. Stewart B. A. (2013). *Principles of Sustainable Soil Management in Agroecosystems.* Boca Raton, FL: CRC Press 10.1201/b14972

[B86] Law I. J. Botha W. J. Majaule U. C. Phalane F. L. (2007). Symbiotic and genomic diversity of ‘cowpea’ bradyrhizobia from soils in Botswana and South Africa. *Biol. Fertil. Soils* 43 653–663. 10.1007/s00374-006-0145-y

[B87] Lemaire B. Van Cauwenberghe J. Chimphango S. Stirton C. Honnay O. Smets E. (2015). Recombination and horizontal transfer of nodulation and ACC deaminase (acdS) genes within Alpha- and Beta-proteobacteria nodulating legumes of the Cape Fynbos biome. *FEMS Microbiol. Ecol.* 91:fiv118. 10.1093/femsec/fiv118 26433010

[B88] Li Y. H. Li W. Zhang C. Yang L. Chang R. Z. Gaut B. S. (2010). Genetic diversity in domesticated soybean (*Glycine max*) and its wild progenitor (*Glycine soja*) for simple sequence repeat and single nucleotide polymorphism loci. *New Phytol.* 188 242–253. 10.1111/j.1469-8137.2010.03344.x 20618914

[B89] Li Y. H. Wang R. Zhang X. X. Young J. P. W. Wang E. T. Sui X. H. (2015). *Bradyrhizobium guangdongense* sp. nov. and *Bradyrhizobium guangxiense* sp. nov., isolated from effective nodules of peanut. *Int. J. Syst. Evol. Microbiol.* 65 4655–4661. 10.1099/ijsem.0.000629 26409482

[B90] Linder H. P. (2003). The radiation of the Cape flora, southern Africa. *Biol. Rev. Camb. Philos. Soc.* 78 597–638. 10.1017/S1464793103006171 14700393

[B91] Lu J. K. Dou Y. J. Zhu Y. J. Wang S. K. Sui X. H. Kang L. H. (2014). Bradyrhizobium ganzhouense sp. nov., an effective symbiotic bacterium isolated from *Acacia melanoxylon* R. Br. nodules. *Int. J. Syst. Evol. Microbiol.* 64 1900–1905. 10.1099/ijs.0.056564-0 24585376 PMC4051118

[B92] Maingi J. M. Gitonga N. M. Shisanya C. A. Hornetz B. Muluvi G. M. (2006). Population levels of indigenous bradyrhizobia nodulating promiscuous soybean in two Kenyan soils of the semi-arid and semi-humid agroecological zones. *J. Agric. Rural Dev. Trop. Subtrop.* 107 149–159.

[B93] Manning J. Goldblatt P. (2012). *Plants of the Greater Cape Floristic Region.* Pretoria: South African National Biodiversity Institute.

[B94] Mapope N. Dakora F. D. (2016). N2 fixation, carbon accumulation, and plant water relations in soybean (*Glycine max* L. Merrill) varieties sampled from farmers’ fields in South Africa, measured using 15N and 13C natural abundance. *Agri. Ecosys. Environ.* 221 174–186. 10.1016/j.agee.2016.01.023

[B95] Michel D. C. Passos S. R. Simões-Araujo J. L. Baraúna A. C. da Silva K. Parma M. M. (2017). *Bradyrhizobium centrolobii* and *Bradyrhizobium macuxiense* sp. nov. isolated from *Centrolobium paraense* grown in soil of Amazonia, Brazil. *Arch. Microbiol.* 199 657–664. 10.1007/s00203-017-1340-y 28180951

[B96] Mohale K. C. Belane A. K. Dakora F. D. (2013). Symbiotic N nutrition, C assimilation, and plant water use efficiency in *Bambara groundnut* (*Vigna subterranea* L. Verdc) grown in farmers’ fields in South Africa, measured using 15N and 13C natural abundance. *Biol. Fertil. Soils* 50 307–319. 10.1007/s00374-013-0841-3

[B97] Mohammed M. Jaiswal S. K. Dakora F. D. (2018). Distribution and correlation between phylogeny and functional traits of cowpea (*Vigna unguiculata* L. Walp.)-nodulating microsymbionts from Ghana and South Africa. *Sci. Rep.* 8:18006. 10.1038/s41598-018-36324-0 30573737 PMC6302100

[B98] Mokgehle S. N. Dakora F. D. Mathews C. (2014). Variation in N2 fixation and N contribution by 25 groundnut (*Arachis hypogaea* L.) varieties grown in different agro-ecologies, measured using 15N natural abundance. *Agri. Ecosyst. Environ.* 195 161–172. 10.1016/j.agee.2014.05.014

[B99] Molouba F. Lorquin J. Willems A. Hoste B. Giraud E. Dreyfus B. (1999). Photosynthetic bradyrhizobia from *Aeschynomene* spp. are specific to stem-nodulated species and form a separate 16S ribosomal DNA restriction fragment length polymorphism group. *Appl. Environ. Microbiol.* 65 3084–3094. 10388707 10.1128/aem.65.7.3084-3094.1999 PMC91460

[B100] Mpepereki S. Javaheri F. Davis P. Giller K. E. (2000). Soyabeans and sustainable agriculture: promiscuous soyabeans in southern Africa. *Field Crops Res.* 65 137–149. 10.1016/S0378-4290(99)00083-0

[B101] Mpepereki S. Wollum A. G. Makonese F. (1996). Diversity in symbiotic specificity of cowpea rhizobia indigenous to Zimbabwean soils. *Plant Soil* 186 167–171. 10.1007/BF00035071

[B102] Musiyiwa K. Mpepereki S. Giller K. E. (2005). Symbiotic effectiveness and host ranges of indigenous rhizobia nodulating promiscuous soyabean varieties in Zimbabwean soils. *Soil Biol. Biochem.* 37 1169–1176. 10.1016/j.soilbio.2004.12.004

[B103] Mutegi J. Zingore S. I. (2014). *Boosting Soybean Production for Improved Food Security and Incomes in Africa.* Nairobi: The International Plant Nutrition Institute (IPNI).

[B104] Naab J. B. Chimphango S. M. Dakora F. D. (2009). N2 fixation in cowpea plants grown in farmers’ fields in the Upper West Region of Ghana, measured using 15N natural abundance. *Symbiosis* 48 37–46. 10.1007/BF03179983

[B105] Naamala J. Jaiswal S. K. Dakora F. D. (2016). Microsymbiont diversity and phylogeny of native bradyrhizobia associated with soybean (*Glycine max* L. Merr.) nodulation in South African soils. *Syst. Appl. Microbiol.* 39 336–344. 10.1016/j.syapm.2016.05.009 27324571 PMC4958686

[B106] Ncube B. Twomlow S. J. van Wijk M. T. Dimes J. P. Giller K. E. (2007). Productivity and residual benefits of grain legumes to sorghum under semi-arid conditions in south western Zimbabwe. *Plant Soil* 299 1–15. 10.1007/s11104-007-9330-5

[B107] Ndungu S. M. Messmer M. M. Ziegler D. Gamper H. A. Mészáros É Thuita M. (2018). Cowpea (*Vigna unguiculata* L. Walp) hosts several widespread bradyrhizobial root nodule symbionts across contrasting agro-ecological production areas in Kenya. *Agric. Ecosyst. Environ.* 261 161–171. 10.1016/j.agee.2017.12.014 29970945 PMC5946706

[B108] Ngo Nkot L. Krasova-Wade T. Etoa F. X. Sylla S. N. Nwaga D. (2008). Genetic diversity of rhizobia nodulating *Arachis hypogaea* L. in diverse land use systems of humid forest zone in Cameroon. *Appl. Soil Ecol.* 40 411–416. 10.1016/j.apsoil.2008.06.007

[B109] Nievas F. Bogino P. Nocelli N. Giordano W. (2012). Genotypic analysis of isolated peanut-nodulating rhizobial strains reveals differences among populations obtained from soils with different cropping histories. *Appl. Soil Ecol.* 53 74–82. 10.1016/j.apsoil.2011.11.010

[B110] Nyemba R. C. Dakora F. D. (2010). Evaluating N2 fixation by food grain legumes in farmers’ fields in three agroecological zones of Zambia, using 15N natural abundance. *Biol. Fertil. Soils* 46 461–470. 10.1007/s00374-010-0451-2

[B111] Nzoué A. Miché L. Klonowska A. Laguerre G. de Lajudie P. Moulin L. (2009). Multilocus sequence analysis of bradyrhizobia isolated from *Aeschynomene* species in Senegal. *Syst. Appl. Microbiol.* 32 400–412. 10.1016/j.syapm.2009.06.002 19556090

[B112] Odee D. W. Haukka K. McInroy S. G. Sprent J. I. Sutherland J. M. Young J. P. W. (2002). Genetic and symbiotic characterization of rhizobia isolated from tree and herbaceous legumes grown in soils from ecologically diverse sites in Kenya. *Soil Biol. Biochem.* 34 801–811. 10.1016/S0038-0717(02)00009-3

[B113] Ojha A. Tak N. Rathi S. Chouhan B. Rao S. R. Barik S. K. (2017). Molecular characterization of novel *Bradyrhizobium* strains nodulating Eriosema chinense and *Flemingia vestita*, Important unexplored native legumes of the Sub-Himalayan region (Meghalaya) of India. *Syst. Appl. Microbiol.* 40 334–344. 10.1016/j.syapm.2017.06.003 28781100

[B114] Okogun J. Sanginga N. (2003). Can introduced and indigenous rhizobial strains compete for nodule formation by promiscuous soybean in the moist savanna agroecological zone of Nigeria? *Biol. Fertil. Soils* 38 26–31. 10.1007/s00374-003-0611-8

[B115] Osei O. Abaidoo R. C. Ahiabor B. D. Boddey R. M. Rouws L. F. (2018). Bacteria related to *Bradyrhizobium yuanmingense* from Ghana are effective groundnut micro-symbionts. *Appl. Soil Ecol.* 127 41–50. 10.1016/j.apsoil.2018.03.003 29887673 PMC5989812

[B116] Osunde A. Gwam S. Bala A. Sanginga N. Okogun J. (2003). Responses to rhizobial inoculation by two promiscuous soybean cultivars in soils of the Southern Guinea savanna zone of Nigeria. *Biol. Fertil. Soils* 37 274–279.

[B117] Paffetti D. Daguin F. Fancelli S. Gnocchi S. Lippi F. Scotti C. (1998). Influence of plant genotype on the selection of nodulating *Sinorhizobium meliloti* strains by *Medicago sativa*. *Antonie Van Leeuwenhoek* 73 3–8. 10.1023/A:1000591719287 9602273

[B118] Parr M. (2014). *Promiscuous Soybean: Impacts on Rhizobia Diversity and Smallholder Malawian Agriculture.* Ph.D. thesis, North Carolina State University Raleigh, NC.

[B119] Peix A. Ramírez-Bahena M. H. Flores-Félix J. D. de la Vega P. A. Rivas R. Mateos P. F. (2015). Revision of the taxonomic status of the species *Rhizobium lupini* and reclassification as *Bradyrhizobium lupini* comb. *IJSEM* 65 1213–1219. 10.1099/ijs.0.000082 25609676

[B120] Peoples M. B. Bergersen F. J. Turner G. L. Sampet C. Rerkasem B. Bhromsiri A. (1991). “Use of the natural enrichment of 15N in plant available soil N for the measurement of symbiotic N2 fixation,” in *Proceedings of the International Symposium on Stable Isotopes in Plant Nutrition, Soil Fertility and Environmental Studies* (Vienna: FAO) 117–129.

[B121] Peoples M. B. Brockwell J. Herridge D. F. Rochester I. J. Alves B. J. R. Urquiaga S. (2009). The contributions of nitrogen-fixing crop legumes to the productivity of agricultural systems. *Symbiosis* 48 1–17. 10.1007/BF03179980

[B122] Polhill R. M. VanWyk B. E. (2005). “Genisteae,” in *Legumes of the World* eds Lewis G. Schrire B. Mackinder B. Lock M. (Richmond, VA: Royal Botanic Gardens, Kew) 283–297.

[B123] Pule-Meulenberg F. Belane A. K. Krasova-Wade T. Dakora F. D. (2010). Symbiotic functioning and bradyrhizobial biodiversity of cowpea (*Vigna unguiculata* L. Walp.) in Africa. *BMC Microbiol.* 10:89. 10.1186/1471-2180-10-89 20331875 PMC2858033

[B124] Pule-Meulenberg F. Dakora F. D. (2009). Assessing the symbiotic dependency of grain and tree legumes on N2 fixation for their N nutrition in five agro-ecological zones of Botswana. *Symbiosis* 48 68–77. 10.1007/BF03179986

[B125] Pulver E. L. Kueneman E. A. Ranga-Rao V. (1985). Identification of promiscuous nodulating soybean efficient in N2 fixation. *Crop Sci.* 25 660–663. 10.2135/cropsci1985.0011183X002500040019x

[B126] Puozaa D. K. Jaiswal S. K. Dakora F. D. (2017). African origin of Bradyrhizobium populations nodulating *Bambara groundnut* (*Vigna subterranea* L. Verdc) in Ghanaian and South African soils. *PLoS One* 12:e0184943. 10.1371/journal.pone.0184943 28945783 PMC5612659

[B127] Ramírez-Bahena M. H. Chahboune R. Peix A. Velázquez E. (2013). Reclassification of Agromonas oligotrophica into the genus Bradyrhizobium as *Bradyrhizobium oligotrophicum* comb. nov. *IJSEM* 63 1013–1016. 10.1099/ijs.0.041897-0 22685107

[B128] Ramírez-Bahena M. H. Flores-Félix J. D. Chahboune R. Toro M. Velázquez E. Peix A. (2016). *Bradyrhizobium centrosemae* (symbiovar centrosemae) sp. nov., *Bradyrhizobium americanum* (symbiovar phaseolarum) sp. nov. and a new symbiovar (tropici) of *Bradyrhizobium viridifuturi* establish symbiosis with *Centrosema* species native to America. *Syst. Appl. Microbiol.* 39 378–383. 10.1016/j.syapm.2016.06.001 27394069

[B129] Ramírez-Bahena M. H. Peix A. Rivas R. Camacho M. Rodriguez-Navarro D. N. Mateos P. F. (2009). *Bradyrhizobium pachyrhizi* sp. nov. and *Bradyrhizobium jicamae* sp. nov., isolated from effective nodules of *Pachyrhizus erosus*. *IJSEM* 59 1929–1934. 10.1099/ijs.0.006320-0 19567584

[B130] Rathi S. Tak N. Bissa G. Chouhan B. Ojha A. Adhikari D. (2018). Selection of Bradyrhizobium or Ensifer symbionts by the native Indian caesalpinioid legume *Chamaecrista pumila* depends on soil pH and other edaphic and climatic factors. *FEMS Microbiol. Ecol.* 94:fiy180. 10.1093/femsec/fiy180 30184201

[B131] Risal C. P. Yokoyama T. Ohkama-Ohtsu N. Djedidi S. Sekimoto H. (2010). Genetic diversity of native soybean bradyrhizobia from different topographical regions along the southern slopes of the Himalayan Mountains in Nepal. *Syst. Appl. Microbiol.* 33 416–425. 10.1016/j.syapm.2010.06.008 20851547

[B132] Rivas R. Willems A. Palomo J. L. García-Benavides P. Mateos P. F. Martínez-Molina E. (2004). *Bradyrhizobium betae* sp. nov., isolated from roots of *Beta vulgaris* affected by tumour-like deformations. *Int. J. Syst. Evol. Microbiol.* 54 1271–1275. 10.1099/ijs.0.02971-0 15280302

[B133] Roughley R. J. (1970). The influence of root temperature, Rhizobium strain and host selection on the structure and nitrogen-fixing efficiency of the root nodules of *Trifolium subterraneum*. *Ann. Bot.* 34 631–646. 10.1093/oxfordjournals.aob.a084397

[B134] Ruben R. Pender J. Kuyvenhoven A. (2007). *Sustainable Poverty Reduction in Less-Favoured Areas.* Wallingford: CABI 10.1079/9781845932770.0000

[B135] Salvagiotti F. Cassman K. G. Specht J. E. Walters D. T. Weiss A. Dobermann A. (2008). Nitrogen uptake, fixation and response to fertiliser N in soybeans: a review. *Field Crops Res.* 108 1–13. 10.1016/j.fcr.2008.03.001

[B136] Sanginga N. Abaidoo R. C. Dashiell K. E. Carsky R. J. Okogun A. (1996). Persistence and effectiveness of rhizobia nodulating promiscuous soybeans in moist savannah zones of Nigeria. *Appl. Soil Ecol.* 3 215–224. 10.1016/0929-1393(95)00089-5

[B137] Sanginga N. Thottappilly G. Dashiell K. (2000). Effectiveness of rhizobia nodulating recent promiscous soybean in the moist savanna of Nigeria. *Soil Biol. Biochem.* 32 127–133. 10.1016/S0038-0717(99)00143-1

[B138] Serraj R. (2004). *Symbiotic Nitrogen Fixation: Prospects for Enhanced Application in Tropical Agriculture.* New York, NY: Science Publishers.

[B139] Sessitsch A. Howieson J. Perret X. Antoun H. Martinez-Romero E. (2002). Advances in Rhizobium research. *Crit. Rev. Plant Sci.* 21 323–378. 10.1080/0735-260291044278

[B140] Shurtleff W. Aoyagi A. (2009). *History of Soybeans and Soyfoods in Africa (1857-2009). Bibliography and Source Book.* Lafayette, CA: Soyinfo Center.

[B141] Siddique K. H. M. Johansen C. Turner N. C. Jeuffroy M. H. Hashem A. Sakar D. (2011). Innovations in agronomy for food legumes. *Agron. Sustain. Dev.* 32 45–64. 10.1007/s13593-011-0021-5

[B142] Silva F. V. De Meyer S. E. Simões-Araújo J. L. Barbé T. C. Xavier G. R. O’Hara G. (2014). *Bradyrhizobium manausense* sp. nov., isolated from effective nodules of *Vigna unguiculata* grown in Brazilian Amazon rainforest soils. *Int. J. Syst. Evol. Microbiol.* 64 2358–2363. 10.1099/ijs.0.061259-0 24744018

[B143] Singh G. (2010). *The Soybean: Botany, Production and Uses.* Wallingford: CABI.

[B144] Sprent J. I. (2009). *Bacteria Nodulating Legumes, in Legume Nodulation: A Global Perspective.* Oxford: Wiley-Blackwell 10.1002/9781444316384

[B145] Sprent J. I. Ardley J. James E. K. (2017). Biogeography of nodulated legumes and their nitrogen-fixing symbionts. *New Phytol.* 215 40–56. 10.1111/nph.14474 28211601

[B146] Sprent J. I. Ardley J. K. James E. K. (2013). From North to South: a latitudinal look at legume nodulation processes. *S. Afr. J. Bot.* 89 31–41. 10.1016/j.sajb.2013.06.011

[B147] Sprent J. I. Odee D. W. Dakora F. D. (2010). African legumes: a vital but under-utilized resource. *J. Exp. Bot.* 61 1257–1265. 10.1093/jxb/erp342 19939887

[B148] Steenkamp E. T. Ste T. Przymusiak A. Botha W. J. Law I. J. Stepkowski T. (2008). Cowpea and peanut in southern Africa are nodulated by diverse *Bradyrhizobium* strains harboring nodulation genes that belong to the large Pantropical clade common in Africa. *Mol. Phylogenet. Evol.* 48 1131–1144. 10.1016/j.ympev.2008.04.032 18539053

[B149] Stȩpkowski T. Moulin L. Krzyżańska A. McInnes A. Law I. J. Howieson J. (2005). European origin of *Bradyrhizobium* populations infecting lupins and serradella in soils of Western Australia and South Africa. *Appl. Environ. Microbiol.* 71 7041–7052. 10.1128/AEM.71.11.7041-7052.2005 16269740 PMC1287703

[B150] Suzuki Y. Adhikari D. Itoh K. Suyama K. (2014). Effects of temperature on competition and relative dominance of *Bradyrhizobium japonicum* and *Bradyrhizobium elkanii* in the process of soybean nodulation. *Plant Soil* 374 915–924. 10.1007/s11104-013-1924-5

[B151] Sylla S. N. Samba R. T. Neyra M. Ndoye I. Giraud E. Willems A. (2002). Phenotypic and genotypic diversity of rhizobia nodulating *Pterocarpus ernaceus* and *P. lucens* in Senegal. *Syst. Appl. Microbiol.* 25 572–583. 10.1078/07232020260517715 12583718

[B152] Tajima R. Abe J. Lee O. N. Morita S. Lux A. (2008). Developmental changes in peanut root structure during root growth and root-structure modification by nodulation. *Ann. Bot.* 101 491–499. 10.1093/aob/mcm322 18256023 PMC2710188

[B153] Taurian T. Ibanez F. Fabra A. Aguilar O. M. (2006). Genetic diversity of rhizobia nodulating *Arachis hypogaea* L. in central Argentinean soils. *Plant Soil.* 282 41–52. 10.1007/s11104-005-5314-5

[B154] Tefera H. (2011). “Breeding for promiscuous soybeans at IITA,” in *Soybean – Molecular Aspects of Breeding* ed. Sudaric A. (London: IntechOpen Limited) 147–163.

[B155] Temprano-Vera F. Rodriguez-Navarro D. N. Acosta-Jurado S. Perret X. Fossou R. K. Navarro-Gómez P. (2018). *Sinorhizobium fredii* strains HH103 and NGR234 form nitrogen fixing nodules with diverse wild soybeans (*Glycine soja*) from central china but are ineffective on Northern China accessions. *Front. Microbiol.* 9:2843. 10.3389/fmicb.2018.02843 30519234 PMC6258812

[B156] Thies J. E. Bohlool B. B. Singleton P. W. (1992). Environmental effects on competition for nodule occupancy between introduced and indigenous rhizobia and among introduced strains. *Can. J. Microbiol.* 38 493–500. 10.1139/m92-081

[B157] Thies J. E. Singleton P. W. Bohlool B. B. (1991). Modeling symbiotic performance of introduced rhizobia in the field by use of indices of indigenous population size and nitrogen status of the soil. *Appl. Environ. Microbiol.* 57 29–37. 16348397 10.1128/aem.57.1.29-37.1991 PMC182660

[B158] Tilman D. Balzer C. Hill J. Befort B. L. (2011). Global food demand and the sustainable intensification of agriculture. *Proc. Nat. Acad. Sci.* 108 20260–20264. 10.1073/pnas.1116437108 22106295 PMC3250154

[B159] Toomsan B. McDonagh J. F. Limpinuntana V. J. H. A. Giller K. E. (1995). Nitrogen fixation by groundnut and soyabean and residual nitrogen benefits to rice in farmers’ fields in Northeast Thailand. *Plant Soil.* 175 45–56. 10.1007/BF02413009

[B160] Unkovich M. Herridge D. Peoples M. Cadisch G. Boddey B. Giller K. (2008). *Measuring Plant-Associated Nitrogen Fixation in Agricultural Systems.* Canberra: Australian Centre for International Agricultural Research (ACIAR).

[B161] Van Berkum P. Leibold J. M. Eardly B. D. (2006). Proposal for combining *Bradyrhizobium* spp.(*Aeschynomene indica*) with *Blastobacter denitrificans* and to transfer *Blastobacter denitrificans* (Hirsch and Muller, 1985) to the genus Bradyrhizobium as *Bradyrhizobium denitrificans* (comb. nov.). *Syst. Appl. Microbiol.* 29 207–215. 10.1016/j.syapm.2005.07.014 16564957

[B162] van Vugt D. Franke A. C. Giller K. E. (2018). Understanding variability in the benefits of N2-fixation in soybean-maize rotations on smallholder farmers’ fields in Malawi. *Agric. Ecosyst. Environ.* 261 241–250. 10.1016/j.agee.2017.05.008 29970952 PMC5946708

[B163] Vinuesa P. Leon-Barrios M. Silva C. Willems A. Jarabo-Lorenzo A. Perez- Galdona R. (2005). *Bradyrhizobium canariense* sp. nov., an acid-tolerant endosymbiont that nodulates endemic genistoid legumes (Papilionoideae: Genisteae) from the Canary Islands, along with *Bradyrhizobium japonicum* bv. genistearum, *Bradyrhizobium* genospecies alpha and Bradyrhizobium genospecies beta. *Int. J. Syst. Evol. Microbiol.* 55 569–575. 10.1099/ijs.0.63292-0 15774626

[B164] Vitousek P. M. Menge D. N. Reed S. C. Cleveland C. C. (2013). Biological nitrogen fixation: rates, patterns and ecological controls in terrestrial ecosystems. *Philos. Trans. R. Soc. B Biol. Sci.* 368:20130119. 10.1098/rstb.2013.0119 23713117 PMC3682739

[B165] Wade T. K. Le Quéré A. Laguerre G. N’zoué A. Ndione J. A. Dorego F. (2014). Eco-geographical diversity of cowpea bradyrhizobia in Senegal is marked by dominance of two genetic types. *Syst. Appl. Microbiol.* 37 129–139. 10.1016/j.syapm.2013.10.002 24373721

[B166] Wang J. Y. Wang R. Zhang Y. M. Liu H. C. Chen W. F. Wang E. T. (2013). *Bradyrhizobium daqingense* sp. nov., isolated from soybean nodules. *Int. J. Syst. Evol. Microbiol.* 63 616–624. 10.1099/ijs.0.034280-0 22544787

[B167] Wang R. Chang Y. L. Zheng W. T. Zhang D. Zhang X. X. Sui X. H. (2013). *Bradyrhizobium arachidis* sp. nov., isolated from effective nodules of *Arachis hypogaea* grown in China. *Syst. Appl. Microbiol.* 36 101–105. 10.1016/j.syapm.2012.10.009 23295123

[B168] Wani S. Rupela O. Lee K. (1995). “Sustainable agriculture in the semi-arid tropics through biological nitrogen fixation in grain legumes,” in *Management of Biological Nitrogen Fixation for the Development of More Productive and Sustainable Agricultural Systems* eds Ladha J. K. Peoples M. B. (Berlin: Springer) 29–49. 10.1007/978-94-011-0055-7_2

[B169] Witkowski E. T. F. Mitchell D. T. (1987). Variations in soil phosphorus in the fynbos biome, South Africa. *J. Ecol.* 75 1159–1171. 10.2307/2260320

[B170] Wolde-meskel E. Terefework Z. Lindström K. Frostegård Å (2004). Metabolic and genomic diversity of rhizobia isolated from field standing native and exotic woody legumes in southern Ethiopia. *Syst. Appl. Microbiol.* 27 603–611. 10.1078/0723202041748145 15490562

[B171] Xu L. M. Ge C. Cui Z. Li J. Fan H. (1995). *Bradyrhizobium liaoningense* sp. nov., isolated from the root nodules of soybeans. *IJSEM* 45 706–711. 10.1099/00207713-45-4-706 7547289

[B172] Yao Y. Sui X. H. Zhang X. X. Wang E. T. Chen W. X. (2015). *Bradyrhizobium erythrophlei* sp. nov. and *Bradyrhizobium ferriligni* sp. nov., isolated from effective nodules of *Erythrophleum fordii*. *Int. J. Syst. Evol. Microbiol.* 65 1831–1837. 10.1099/ijs.0.000183 25754551

[B173] Yao Z. Y. Kan F. L. Wang E. T. Wei G. H. Chen W. X. (2002). Characterization of rhizobia that nodulate legume species of the genus Lespedeza and description of *Bradyrhizobium yuanmingense* sp. nov. *IJSEM* 52 2219–2230. 12508891 10.1099/00207713-52-6-2219

[B174] Youseif S. H. El-Megeed F. H. A. Khalifa M. A. Saleh S. A. (2014). Symbiotic effectiveness of Rhizobium (Agrobacterium) compared to Ensifer (Sinorhizobium) and Bradyrhizobium genera for soybean inoculation under field conditions. *Res. J. Microbiol.* 9 151–162. 10.3923/jm.2014.151.162

[B175] Yu X. Cloutier S. Tambong J. T. Bromfield E. S. (2014). *Bradyrhizobium ottawaense* sp. nov., a symbiotic nitrogen fixing bacterium from root nodules of soybeans in Canada. *IJSEM* 64 3202–3207. 10.1099/ijs.0.065540-0 24969302 PMC4156109

[B176] Zahran H. H. (1999). Rhizobium-legume symbiosis and nitrogen fixation under severe conditions and in an arid climate. *Microbiol. Mol. Boil. Rev.* 63 968–989. 10585971 10.1128/mmbr.63.4.968-989.1999 PMC98982

[B177] Zhang H. Prithiviraj B. Charles T. C. Driscoll B. T. Smith D. L. (2003). Low temperature tolerant *Bradyrhizobium japonicum* strains allowing improved nodulation and nitrogen fixation of soybean in a short season (cool spring) area. *Eur. J. Agron.* 19 205–213. 10.1016/S1161-0301(02)00038-2

[B178] Zhang Y. M. Li Y. Jr. Chen W. F. Wang E. T. Sui X. H. Li Q. Q. (2012). *Bradyrhizobium huanghuaihaiense* sp. nov., an effective symbiotic bacterium isolated from soybean (*Glycine max* L.) nodules. *Int. J. Syst. Evol. Microbiol.* 62 1951–1957. 10.1099/ijs.0.034546-0 22003042

[B179] Zilli J. E. Baraúna A. C. da Silva K. De Meyer S. E. Farias E. N. Kaminski P. E. (2014). *Bradyrhizobium neotropicale* sp. nov., isolated from effective nodules of *Centrolobium paraense*. *IJSEM* 64 3950–3957. 10.1099/ijs.0.065458-0 25205796

